# Differentiation Driven Changes in the Dynamic Organization of Basal Transcription Initiation

**DOI:** 10.1371/journal.pbio.1000220

**Published:** 2009-10-20

**Authors:** Giuseppina Giglia-Mari, Arjan F. Theil, Pierre-Olivier Mari, Sophie Mourgues, Julie Nonnekens, Lise O. Andrieux, Jan de Wit, Catherine Miquel, Nils Wijgers, Alex Maas, Maria Fousteri, Jan H. J. Hoeijmakers, Wim Vermeulen

**Affiliations:** 1 Department of Genetics, Erasmus MC, Rotterdam, The Netherlands; 2 CNRS, IPBS (Institut de Pharmacologie et de Biologie Structurale), Toulouse, France; 3 Université de Toulouse, UPS, IPBS, Toulouse, France; 4 Department of Toxicogenetics, LUMC, Leiden, The Netherlands; National Cancer Institute, United States of America

## Abstract

Studies based on cell-free systems and on in vitro–cultured living cells support the concept that many cellular processes, such as transcription initiation, are highly dynamic: individual proteins stochastically bind to their substrates and disassemble after reaction completion. This dynamic nature allows quick adaptation of transcription to changing conditions. However, it is unknown to what extent this dynamic transcription organization holds for postmitotic cells embedded in mammalian tissue. To allow analysis of transcription initiation dynamics directly into living mammalian tissues, we created a knock-in mouse model expressing fluorescently tagged TFIIH. Surprisingly and in contrast to what has been observed in cultured and proliferating cells, postmitotic murine cells embedded in their tissue exhibit a strong and long-lasting transcription-dependent immobilization of TFIIH. This immobilization is both differentiation driven and development dependent. Furthermore, although very statically bound, TFIIH can be remobilized to respond to new transcriptional needs. This divergent spatiotemporal transcriptional organization in different cells of the soma revisits the generally accepted highly dynamic concept of the kinetic framework of transcription and shows how basic processes, such as transcription, can be organized in a fundamentally different fashion in intact organisms as previously deduced from in vitro studies.

## Abstract 2

A novel mouse model reveals that the dynamic behavior of transcription factors can vary considerably between different cells of an organism.

## Author Summary

The accepted model of eukaryotic mRNA production is that transcription factors spend most of their time diffusing throughout the cell nucleus, encountering gene promoters (their substrate) in a random fashion and binding to them for a very short time. A similar modus operandi has been accepted as a paradigm for interactions within most of the chromatin-associated enzymatic processes (transcription, replication, DNA damage response). However, it is not known whether such behavior is indeed a common characteristic for all cells in the organism. To answer this question, we generated a knock-in mouse that expresses in all cells a fluorescently tagged transcription factor (TFIIH) that functions in both transcription initiation and DNA repair. This new tool, when combined with quantitative imaging techniques, allowed us to monitor the mobility of this transcription factor in virtually all living tissues. In this study, we show that, in contrast to the aforementioned paradigm, in highly differentiated postmitotic cells such as neurons, hepatocytes, and cardiac myocytes, TFIIH is effectively immobilized on the chromatin during transcription, whereas in proliferative cells, TFIIH has the same dynamic behavior as in cultured cells. Our study also points out that results obtained from in vitro or cultured cell systems cannot always be directly extrapolated to the whole organism. More importantly, this raises a question for researchers in the transcription field: why do some cells opt for a dynamic framework for transcription, whereas others exhibit a static one?

## Introduction

Basal transcription/repair factor IIH (TFIIH) is a ten-subunit complex [1], essential for both RNA polymerase I and II (RNAP1 and 2) transcription initiation and nucleotide excision repair (NER) [2]. NER is an important DNA repair process, which is able to remove a broad spectrum of different DNA lesions. Inherited defects in NER cause severe cancer predisposition and/or premature aging, illustrating its biological significance [3]. In RNAP2 transcription and DNA repair, TFIIH acts as a DNA helix opener, required for transition of initiation to early elongation of RNAP2 and establishment of the preincision NER complex [4],[5]. Mutations in this complex are associated with a surprising phenotypic heterogeneity, ranging from the (skin) cancer-prone disorder xeroderma pigmentosum (XP) to the severe progeroid conditions Cockayne syndrome (CS) and trichothiodistrophy (TTD), the latter additionally characterized by brittle hair and nails [1],[6]–[8]. Since TFIIH is considered to be a general or basal transcription factor and essential NER component, it is surprising to note that TFIIH-associated syndromes present different pathologically affected tissues. For instance, within TTD, primarily differentiated cells appear to be affected. TTD-specific scaly skin and brittle hair features derive from defects in the latest stage of differentiating keratinocytes [9],[10]. In addition, reduced β-globin expression and subsequent anemia in TTD originates from a defective terminal differentiation of precursor erythrocytes [11]. Furthermore, both TTD and XP/CS patients and mice express neurological features caused by defects in final-stage differentiated postmitotic neuronal cells [9],[12]. Nevertheless, many other tissues/organs and cell types appear to be relatively unaffected. This observation can be partly explained by the hypothesis that some tissues are more susceptible than others to endogenous DNA damage [13],[14] and/or that TFIIH transcriptional function is differentially regulated in distinct cell types [15].

Most transcription factors, including basal factors and transcription activators, are only very transiently bound, on the order of a few seconds, to their substrate [16],[17]. This uniformly emerging concept of dynamic transient machineries, with the exception of components of the actual RNAP2 [18], is thought to have a number of advantages over previous proposed models based on stable preassembled large MW “holo” complexes. Live-cell protein mobility studies have culminated in unprecedented, novel insights into the spatial and dynamic organization of macromolecule machines within the context of the complex mammalian cell nucleus with a general, but not universal, modus operandi of dynamic exchange of reaction constituents (proteins). Exceptions to this general mechanism of action have been described for transcriptional activators, such as Gal4 [19] and hypoxia-inducible factor 1 (HIF) [20], and for molecular chaperones such as heat-shock protein 70 [21]. It was found that upon transcription activation these factors reside longer at the transcription sites. Interestingly, the basal transcription factor RNAP2 (GFP-RPB3) was also shown to be longer bound at transcribed regions when transcription was induced [22],[23]. However, it is currently not known whether the generally observed dynamic kinetic framework in in situ–cultured cell types for transcription initiation factors, such as TFIIH [24], can be extrapolated to other cell types in the organisms, under ground-state transcriptional conditions.

To study the consequences of different transcriptional programs on the kinetic behavior of TFIIH and further understand the complex phenotypic expression of mutated TFIIH, we created a knock-in mouse model (Xpb^y/y^) that expresses homozygously a YFP (yellow fluorescent protein, a variant of the green fluorescent protein)-tagged TFIIH subunit under control of the endogenous transcriptional regulatory elements. Using this tool, we explored TFIIH binding kinetics directly in different cells and tissues of the organism.

## Results

### Generation of the XPB-YFP Knock-In Mouse Model

**Figure 1:**
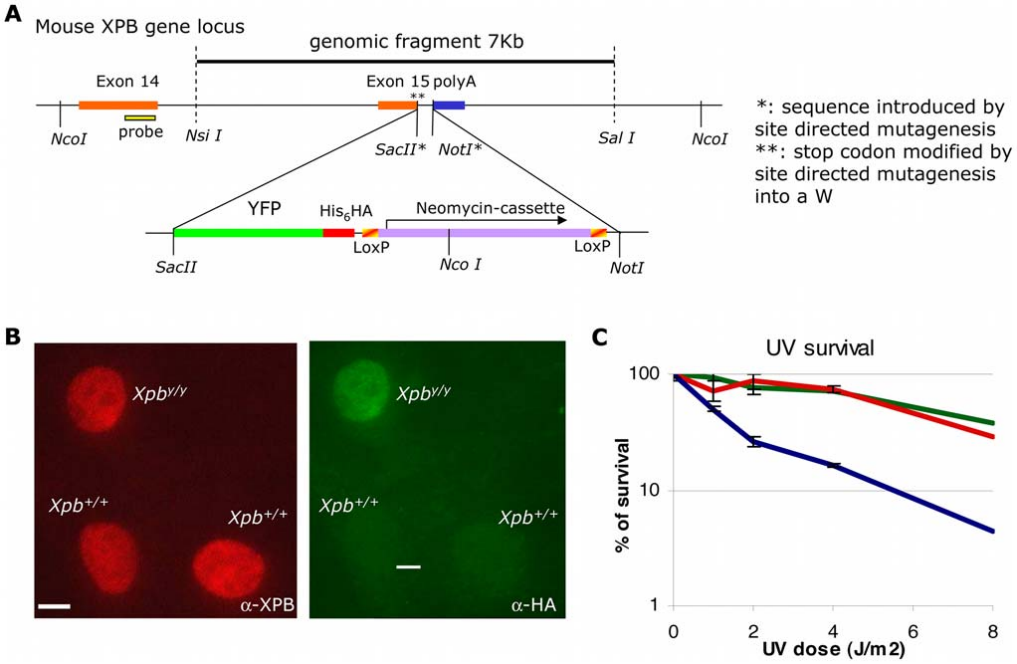
Generation and characterization of a mouse model expressing XPB-YFP^His−HA^ fused protein. (A) Schematic presentation of the 3′ part of the mouse Xpb gene locus, including the last exons 14 and 15. The dotted line indicates the 7-kb mouse genomic DNA fragment used for gene targeting. The translational stop codon (in exon 15) was mutated to allow in-frame fusion with the modified fluorescent marker, YFP (i.e., ATG-less YFP, with an additional stretch of six histidines and an HA epitope tags). As a dominant selectable marker, a neomycin cassette (flanked by two LoxP sites) was introduced downstream the YFP sequence. Details of the targeting strategy are presented in Materials and Methods. (B) Percentage of surviving cells, 48 h after UV irradiation at the indicated doses of dermal fibroblasts isolated from *Xpb^+/+^* mice (wild-type [wt], green), *Xpb^y/y^* mice (red), and *Xpb^y/y^* mice crossed with *Xpc* (NER-deficient) mice (blue). (C) Comparative immunofluorescence of a mixed population of dermal fibroblasts isolated from an untargeted mouse (*Xpb^+/+^*, HA negative) and a targeted mouse (*Xpb^y/y^*, HA positive), stained with anti-XPB (red, left) or anti-HA (green, right), which recognizes the XPB-YFP-His_6_HA protein. Bar: 10 µm.

In order to visualize and quantitatively determine dynamic interactions of transcription initiation factor TFIIH within different postmitotic and differentiated cells and living tissues, we created a mouse knock-in model that expresses the XPB protein (largest subunit and helicase of the ten-subunit TFIIH complex) tagged at its C-terminus with the yellow GFP variant (YFP). Gene-targeting constructs and strategy are schematically depicted in Figure 1A (see Materials and Methods and Figure S1A for details). Briefly, the targeting strategy was designed in such a way that interference of the genomic organization of the *Xpb* locus was kept to a minimum, keeping the integrity of the promoter, all the intron–exon boundaries, and the 3′ UTR (including the endogenous poly A signals) of the *Xpb* gene intact. The XPB-YFP fusion protein further contains additional convenient C-terminal His_6_- and HA-tags. Embryonic stem (ES) cells transfected with the targeting fusion construct were selected for proper targeting (by homologous recombination) by Southern blotting (Figure S1A). Immunoblotting of whole-cell extracts revealed that an intact XPB-YFP fusion protein was produced in recombinant ES cells (Figure S1B). Selected ES cells were introduced in C57Bl/6 blastocysts and transplanted into foster mothers. Chimeric offspring were further crossed for germ-line transmission of the targeted allele (Figure S1D and S1E).

To avoid any possible interference with expression of the fusion gene by the presence of the dominant selectable NeoMarker in the 3′ UTR, heterozygous *Xpb^y−Neo/+^* mice were crossed with a ubiquitous Cre-Recombinase–expressing mouse model [25] (Figure S1D). The subsequent “floxed” heterozygous offspring (*Xpb^y/+^*) were intercrossed to generate homozygous knock-in mice (*Xpb^y/y^*) (Figure S1E). Homozygous *Xpb^y/y^*, heterozygous *Xpb^y/+^*, and wild-type (*Xpb^+/+^*) progeny were obtained in a Mendelian ratio (32 homozygous knock-in mice out of 125 offspring), indicating that homozygosity for the knock-in fusion gene does not impair embryonic development. A small cohort of homozygous (males and females) and heterozygous (six of each) littermates was allowed to age, until natural death, which occurred around 2 years for both *Xpb^y/y^* and *Xpb^y/+^* mice. No obvious features of premature aging or spontaneous carcinogenesis of the *Xpb^y/y^* other than those occurring in *Xpb^+/+^* mice were observed. Knock-in *Xpb^y/y^* mice appeared healthy and fertile, indicating that the presence of the fluorescent tag does not significantly interfere with the vital functions (transcription initiation) of the *Xpb* gene.

Most viable, naturally occurring TFIIH mutations cause an overall reduction of the steady-state levels of TFIIH [26]–[28]. However, comparative immunofluorescence revealed that the intracellular concentration of p62 (Figure S2A) (another nontagged TFIIH subunit) and XPB were not altered by the presence of the tagged XPB subunit (Figure 1B) [26]–[28], suggesting that neither the expression nor the stability was affected by the presence of the YFP-His6_HA tag on the XPB protein. Unscheduled DNA repair synthesis capacity (UDS, a measure of NER activity) after UV damage (Figure S2B) and UV-survival of *Xpb^y/y^* dermal fibroblasts were similar to wild-type (*Xpb^+/+^*) cells assayed in parallel (Figure 1C), indicating that the tagged XPB protein remains normally active in NER. Immunoprecipitation experiments also showed that the tagged XPB protein was incorporated into TFIIH (unpublished data), consistent with our previous observations that exogenously expressed GFP-tagged XPB is properly incorporated into TFIIH complexes [24].

In conclusion, the addition of the 27-kDa fluorescent tag to the strongly conserved XPB protein does not detectably affect the multiple functions of TFIIH in transcription and NER even at the critical level of an intact organism, whereas single amino acid substitutions in *XPB* patients give rise to severe skin cancer predisposition and dramatic premature aging [29],[30]. This demonstrates that the *Xpb^y/y^* knock-in mouse model is a bona fide source to obtain relevant information on the spatial and dynamic organization of transcription and DNA repair in vivo in an intact organism. Fluorescence of TFIIH was detectable in all primary cultures of different cell types isolated from these mice, e.g., ES cells, dermal fibroblasts, and keratinocytes (Figure S2C). Note that the level and subnuclear distribution of fluorescence throughout the cell population is homogeneous (Figure S2C), in striking contrast to the heterogeneous expression characteristic of stably transfected cell cultures.

### TFIIH Mobility Is Different in Distinct Living Tissues

**Figure 2:**
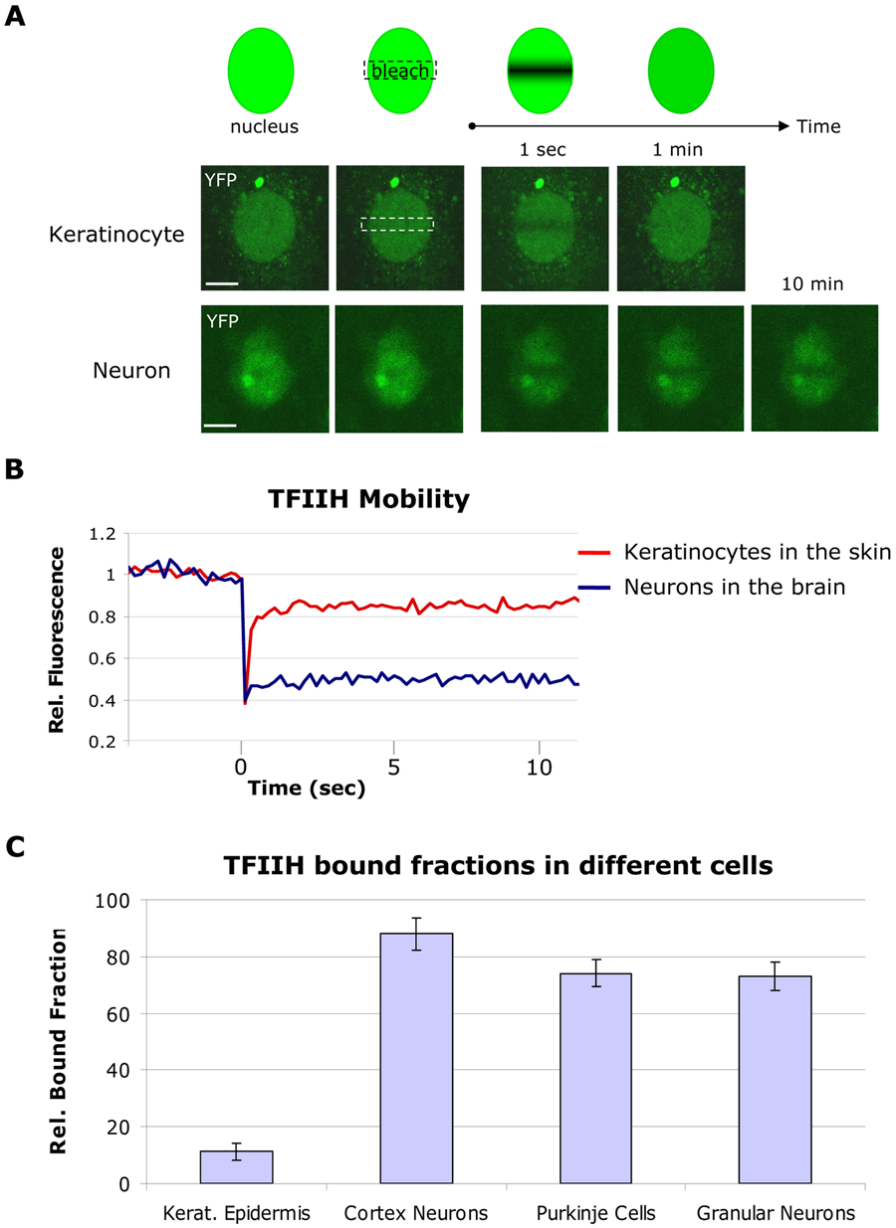
Mobility of TFIIH in cultured cells and in living tissues. (A) FRAP assay (upper panel) and confocal images of keratinocytes (middle panel) and cortex neurons (lower panel) within tissue sections during FRAP analysis. A small region in the middle of the nucleus is bleached; the subsequent recovery is followed in time for 10 min. Fluorescence is quickly recovered in keratinocytes (less than 10 s; see Figure 2B), whereas in neurons after 10 min, fluorescence in the bleached area is still not fully recovered. Bar: 20 µm. (B) FRAP graph in which the relative fluorescence (Rel. Fluorescence) recovery after bleaching is plotted against time (seconds). The recovery curve of keratinocytes is shown in red and the curve from cortex neurons in blue. (C) TFIIH bound fractions in different cells, calculated as described in Materials and Methods. Amount of immobilized TFIIH is indicated as percentage of the total amount of TFIIH. Error bars represent the standard error of the mean. Rel.: relative.

To study the spatiotemporal distribution of TFIIH and to determine its kinetic engagements in different cell types within intact tissues, we established organotypic cultures of several organs and tissues. Within organotypic slices of cerebral cortex, isolated and maintained according to established procedures [31], the different cortex layers and the neurons are easily recognizable (Figure S3A). We determined live-cell protein mobility of TFIIH by fluorescence recovery after photobleaching (FRAP) (see Figure 2A, top panel, and Materials and Methods). Using exogenously expressed XPB-GFP, we previously demonstrated that TFIIH in SV40-immortalized human fibroblasts is highly dynamic: the majority moves freely through the nucleus, and a fraction transiently interacts with promoters for only a few seconds (2–6 s) [24]. We found a similar high mobility in primary keratinocytes when monitored within the epidermis of skin explants from *Xpb* ^y/y^ mice (Figure 2A, middle panel), where fluorescence fully recovered in the bleached strip within a few seconds. In sharp contrast, FRAP on neurons within cerebral cortex slices revealed a striking incomplete fluorescence recovery, even after 60 min postbleaching (Figure 2A, lower panel, and Figure S3B), indicating an unprecedented large (>80%) immobile pool of TFIIH (Figure 2B) stably bound to static nuclear structures, most likely chromatin. This unexpected static behavior of a transcription initiation factor, which can be compared to the static behavior of H2B in cultured cells [32], was also observed in Purkinje cells and cerebellar granular neurons in organotypic slices (Figure 2C and Figure S4) suggesting that this behavior is a common feature in various neurons, despite their different functions, chromatin compaction, and different TFIIH expression levels (Figure S5A). Moreover, computation of the (squared) Pearson product moment correlation coefficient between measured immobile fractions and single-cell TFIIH expression levels showed no significant linear relation between these parameters (*r* ^2^ = 0.062). We also measured the mobility of nonfused GFP in neurons within organotypic cerebellar slices derived from a mouse that expressed GFP under the control of actin promoter [33]. In contrast to TFIIH mobility, GFP itself diffuses very rapidly in neurons (Figure S5B), as was previously found in cultured cells [34],[35]. These results indicate that this static behavior is specific for TFIIH and not a common phenomenon of nuclear protein mobility in neurons.

### Mobility of TFIIH in Neurons Reflects TFIIH Cellular Engagement in Transcription

**Figure 3:**
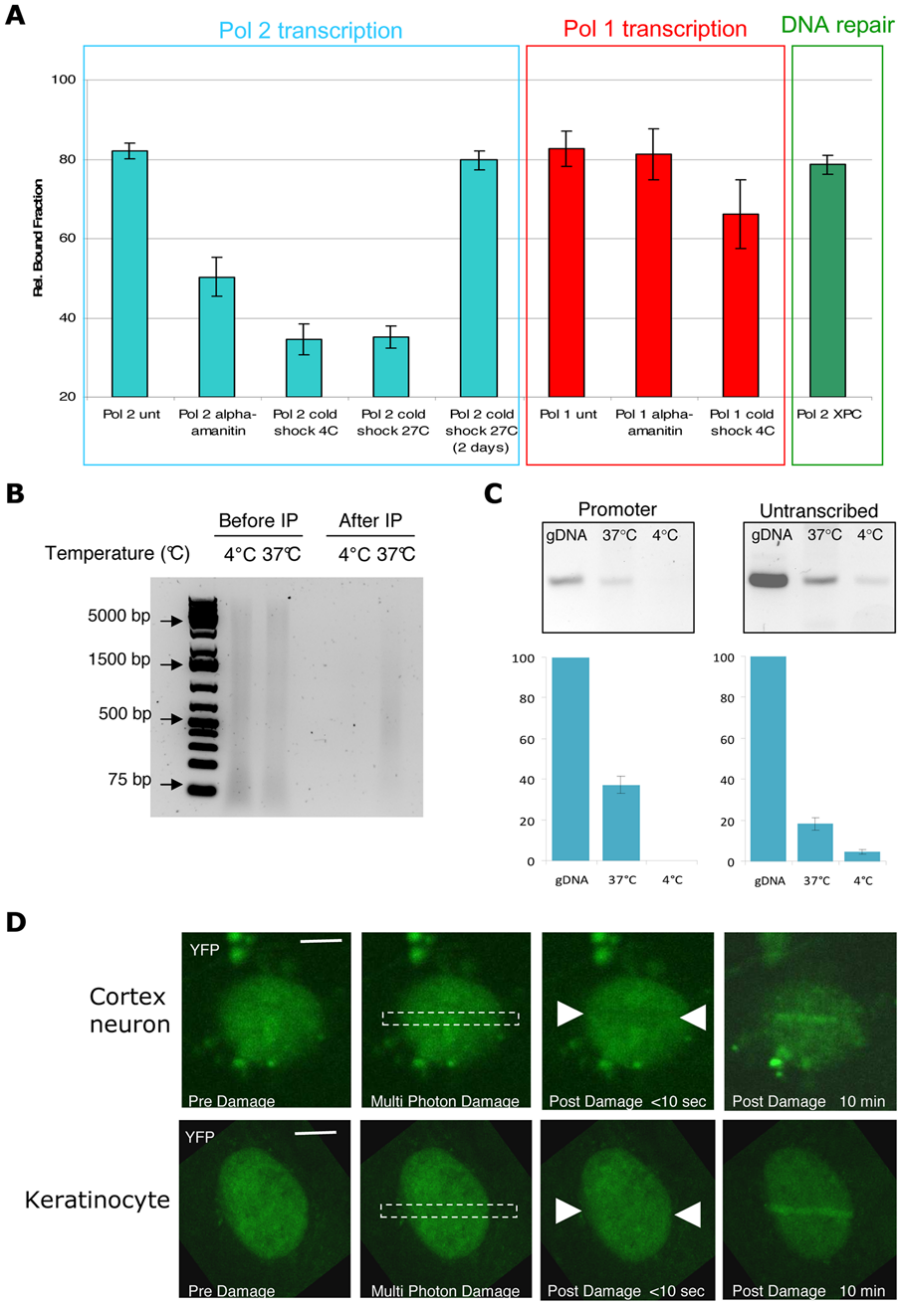
Mobility of TFIIH in cortex neurons during transcription and repair. (A) TFIIH bound fractions in cortex neurons from Xpb^y/y^ mice treated with α-amanitin (transcription inhibition) or submitted to a cold-shock treatment (4°C or 27°C) for 1 h and 2 d (27°C); the effect on TFIIH mobility was compared to the untreated samples (unt). To measure TFIIH dynamics during Pol 2 transcription, measurements were conducted in the nucleoplasm (blue square); to measure TFIIH dynamics during Pol 1 transcription, measurements were conducted in the nucleoli (red square). TFIIH bound fractions were measured in cortex neurons from Xpb^y/y^•Xpc^−/−^ (NER-deficient) mice (green square). Error bars represent the standard error of the mean. Rel.: relative. (B) Equal amounts of sonicated chromatin solution were analyzed on SYBR Green–stained agarose gel after proteinase K treatment and reversal of the cross-links (lane 1 and 2). Following sonication, HA-antibody was added to the chromatin solution for immunoprecipitation of TFIIH complex. Equal volume of immunoprecipitated DNA fraction complex was loaded on gel (lane 3 and 4). (C) Semiquantitative PCR on 200 ng of genomic DNA (gDNA) and 200 ng of HA-precipitated chromatin from cortex slice incubated at 37°C and 4°C. Promoter sequence from the XPB gene (−143/−274) and untranscribed region adjacent to the XPB gene (chr 18: 32479000–32478600) were amplified. PCR products were quantified and plotted on a bar graph: the y-axis represents the percentage of amplified sequences from the HA-precipitated chromatin versus amplified sequences from gDNA. Asterisk (*): undetectable. Error bars have been calculated for three biological replicates. (D) TFIIH accumulation after multiphoton laser induction of DNA damage in cortex neurons (upper panel) versus cultured keratinocytes (lower panel). Images are taken before and after the damage at different indicated time points. Bar: 10 µm.

How does TFIIH immobilization relate to its multiple biological activities? Obviously, the engagement of TFIIH in transcription is most relevant in tissue sections not treated with DNA-damaging agents. However, to exclude that an eventual NER-dependent binding activity could account for the immobilized TFIIH in neurons, initiated by a possible high load of endogenously produced lesions, we measured TFIIH mobility in NER-deficient mice. For this purpose, we crossed Xpb^y/y^ mice with Xpc^−/−^, to generate Xpb^y/y^•Xpc^−/−^ mice. In the absence of XPC, TFIIH does not bind damaged DNA [36]. TFIIH mobility in neurons from Xpb^y/y^•Xpc^−/−^ mice appeared identical as in neurons derived from NER-proficient mice (Figure 3A and Figure S6), showing that the DNA repair function of TFIIH is not responsible for the protracted binding of TFIIH. To demonstrate that the transcription function is responsible for TFIIH immobilization, we inhibited transcription by treating organotypic brain slices with the RNAP2-specific transcription inhibitor α-amanitin [37]. α-Amanitin blocks the catalytic domain of RNAP2 [38], inhibiting both transcription initiation and elongation. Treatment of organotypic tissues with α-amanitin resulted in a release of immobilized TFIIH (Figure 3A and Figure S7), suggesting that the immobilization is due to the transcriptional function of TFIIH [17],[24]. In parallel, we verified that the condition used (incubation time and drug concentration) for transcription inhibition in tissues blocked transcription in cultured cells by measuring the BrU incorporation (Figure S8A and S8B). To further prove that the transcriptional engagement of TFIIH causes its high immobilization in neurons, we modulated transcription by inducing a cold shock (4°C or 27°C). As with the heat-shock response, cold shock generally induces a reduction in basal transcription and translation and a growth arrest [39]. This response is temporary, since after an adaptation period, cellular metabolism is resumed, although at a lower rate compared to growth at 37°C [40]. Moreover, after cold shock, a change in global expression of genes is observed [41] to allow adaptation to this environmental stress. As shown in Figure 3A and Figure S7, reducing the temperature of organotypic slices to extreme (4°C) and moderate hypothermia values (27°C) for 30 to 60 min fully remobilized TFIIH in neurons. To check whether this is a temporary stress response, we also measured the mobility of TFIIH in neurons embedded in organotypic slices kept at 27°C for 48 h. Under these conditions, TFIIH was found to bind as in untreated organotypic slices (37°C), demonstrating that the remobilization of TFIIH at low temperatures is a rapid (60 minutes at 4°C or 27°C) temporary response, likely reflecting a change in the transcriptional engagement (Figure 3A and Figure S7).

Additionally to RNAP2 transcription, TFIIH has been found to accumulate in nucleoli and participate in RNAP1 transcription [24],[42]. Although the clear TFIIH function in RNAP1 transcription has not been elucidated yet, dynamic studies showed that TFIIH residence time in RNAP2 transcription (2–10 s) is different from RNAP1 transcription (∼25 s), suggesting that the role played by TFIIH in these two cellular functions could be slightly different [24]. Cortex neurons and Purkinje cells show a strong localization of TFIIH in nucleoli (Figure S9A), allowing local FRAP analysis in this subnuclear compartment. Similarly to nucleoplasmic TFIIH immobilization (RNAP2 transcription), nucleolar TFIIH immobilization (RNAP1 transcription) was also very high (Figure 3A and Figure S10). In contrast, however, this immobilization is partly resistant to cold shock and resistant to α-amanitin treatment, in line with the expectation, as α-amanitin is known to exclusively inhibit RNAP2 transcription (Figure 3A and Figure S8). To determine that moderate cold shock would indeed inhibit RNAP1 transcription, we measured the amount of pre-rRNA 45S in cold shock–treated cells and tissues (organotypic brain cultures). Surprisingly, cold shock did not alter the amount of 45S, either in cultured cells or in organotypic cortex slices (Figure S9B). The absence of a reduction of the steady-state-levels of 45S rRNA by either a severe (4°C) or a mild (27°C) cold shock is likely explained by the fact that cold shock also interferes with pre-rRNA maturation or degradation. In fact, some proteins, induced by cold shock, have been showed to play a role in preventing the degradation of RNA molecules (reviewed in [43]). In contrast, RNAP1 transcription inhibition induced by actinomycin D (0.1 µg/ml) led to a clear reduction of the amount of pre-rRNA in cultured cells. In conclusion, our results suggest that TFIIH is highly immobilized in neurons in both RNAP2 and RNAP1 transcription.

As TFIIH is known to be involved in RNAP2 transcription initiation, its transcription-dependent immobilization in neurons predicts a favored binding of TFIIH at promoter sequences. To verify that indeed TFIIH in cortex neurons was bound to promoters of active genes, we performed chromatin immunoprecipitation (ChIP) on adult cortex tissues slices under normal conditions and after cold shock, and measured the proportion of active housekeeping genes (*xpb, RnaPolI*) promoter sequences versus adjacent untranscribed areas by semiquantitative PCR (Figure 3C and Figure S9D). As shown by the FRAP experiments (Figure 3A and Figure S7), during cold shock, TFIIH is released from chromatin (Figure 3B). Importantly, we found that TFIIH in cortex slices is more strongly bound to promoter sequences (40% of the input genomic DNA) than to untranscribed areas (19%) (see Materials and Methods for details, Figure 3C and Figure S9D). However, after cold shock, TFIIH is less bound to promoters, clearly showing that cold shock–induced transcription inhibition is associated with remobilization of TFIIH from housekeeping gene promoters.

In view of the notion that the majority of TFIIH is immobilized to chromatin, we wondered whether TFIIH would be available to act in NER after a sudden high dose of genotoxic stress. Since neurons located in the slice are inaccessible to UV-C light, we used multiphoton laser irradiation to locally induce DNA damage [44],[45] in cerebral neurons and compared the accumulation of TFIIH to that observed in skin keratinocytes damaged by the same procedure. Surprisingly, despite the large fraction of immobilized TFIIH, significant amounts of TFIIH were still recruited to damaged DNA (Figure 3D).

### Transcription Organization Varies during Development

**Figure 4:**
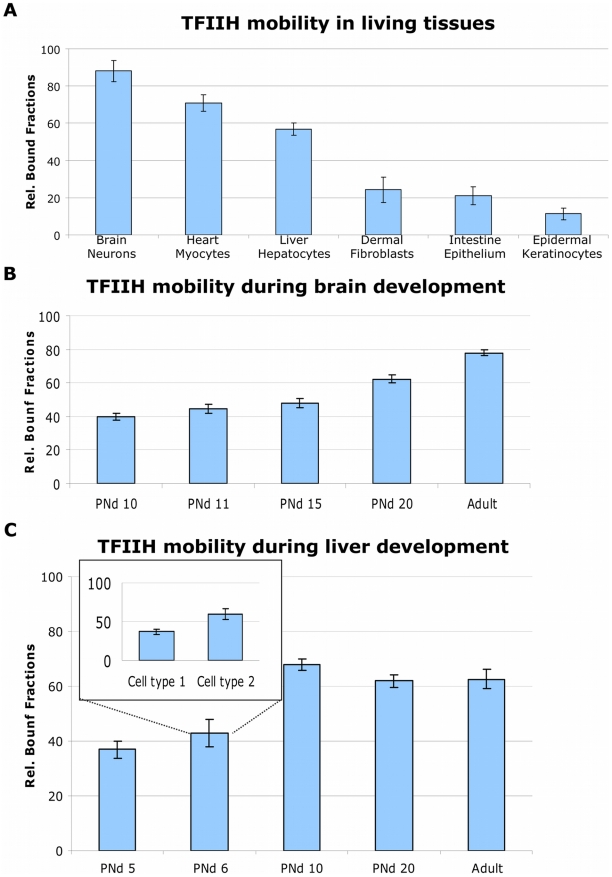
Mobility of TFIIH in different cell types and during development. (A) TFIIH bound fractions are determined in different (indicated) cell types in their tissue. Rel.: relative. (B) TFIIH bound fractions determined at different stages of brain development. PNd, postnatal day. (C) TFIIH bound fractions during different stages of liver development. The insert shows two distinct groups of cells having two different levels of TFIIH immobilization. The error bars represent the standard error of the mean.

Since the observed high TFIIH immobilization is not found in all cell types (Figure 2C), we exploited the availability of an entire organism to analyze the dynamic distribution of TFIIH in different cell types within the context of living tissues (Figure 4A and Figure S11). Specifically in postmitotic and nonproliferative cells (neurons, myocytes, and hepatocytes), we identified a large pool of immobilized TFIIH, whereas in proliferating cells and/or cells that have the capacity to proliferate (intestine epithelium, epidermal keratinocytes, and dermal fibroblasts), TFIIH was found to be highly mobile (Figure 4A and Figure S11). This would suggest that the kinetic organization of this essential transcription factor would be determined by the proliferative capacity of cells. However cultured chondrocytes maintained in a confluent state under low serum were shown to become quiescent (as shown by the absence of the Ki67 marker, Figure S12A) and did not show a difference in TFIIH mobility when compared with proliferative chondrocytes (Figure S12B), suggesting that absence of proliferation is not a condition sufficient to cause a reduction in TFIIH mobility. In view of this result, we investigated whether TFIIH mobility is affected during the establishment of a differentiation-dependent specific transcriptional program. We measured TFIIH mobility during postnatal development of cerebral cortex and liver. Brain and liver were isolated from pups at different postnatal days (PN_d_), and TFIIH mobility was measured in cortex neurons and hepatocytes. Remarkably, we observed a progressive TFIIH immobilization during development (Figure 4B and Figure S13) (Figure 4C and Figure S14), which appeared time- and organ-specific. In cortex neurons, TFIIH bound fractions are gradually increasing from PN_d_ 10 (Figure 4B and Figure S13). TFIIH mobility in neurons is very homogeneous throughout the tested population of cells at each different developmental stage, whereas in liver during development at, e.g., PN_d_ 6, the kinetic pools differ over the population (see inset, Figure 4C) and becomes homogeneous at later stages. Our results demonstrate that the strong TFIIH binding is a physiological event that takes place during normal development of organs and suggests the establishment of a more fixed transcriptional program than in rapidly growing cells.

### Mobility of TFIIH in Differentiated Cells In Vivo and In Vitro

**Figure 5:**
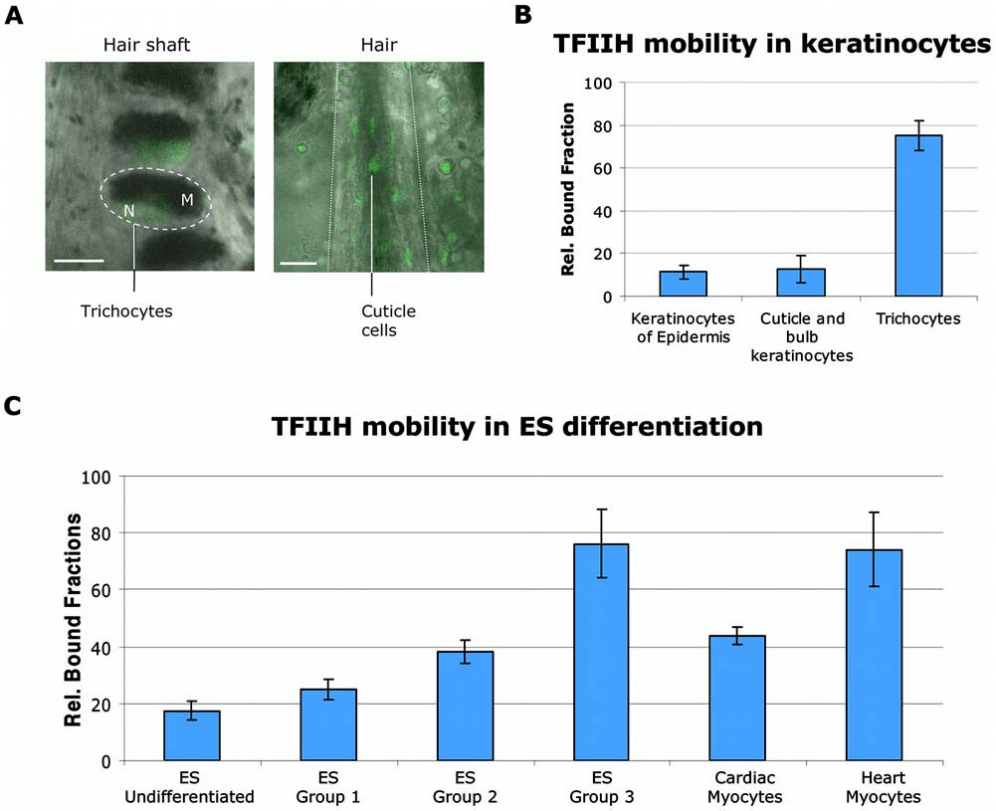
Mobility of TFIIH during in vivo and in vitro differentiation. (A) Left panel: image (bar: 10 µm) of the hair shaft with trichocytes; the dotted circle indicate the body of a trichocyte. Right panel: image of a hair bulb; the dotted line indicates the limit of the hair (bar: 50 µm). Within the hair shaft, cuticle cells are recognizable. M, cytoplasmic melanin inclusions; N, nucleus. (B) TFIIH bound fractions determined in proliferative and differentiated keratinocytes in the tissue. Rel.: relative. (C) TFIIH bound fractions within different populations of in vitro differentiated ES cells and mature heart myocytes within the heart muscle. The error bars represent the standard error of the mean.

To further investigate differentiation-dependent mobility of TFIIH, we used an in vivo keratinocyte differentiation model. Within the hair shaft, highly differentiated nonproliferative keratinocytes, known as trichocytes [46], can be found. These cells produce the keratins and keratin-associated proteins that form the structure of hairs. Trichocytes are easily recognizable because of their position in the hair and of the melanin inclusions in their cytoplasm (Figure 5A, left panel). Indeed, in trichocytes (Figure 5B and Figure S15), TFIIH mobility is greatly reduced, almost to the same extent as in neurons and myocytes, whereas in other keratinocytes of the hair follicle (bulb) (Figure 5A, right panel), TFIIH is highly mobile (Figure 5B and Figure S15). To substantiate that indeed differentiation is an important determinant for the observed shift in TFIIH mobility, we measured TFIIH mobility during in vitro differentiation. ES cells were nonspecifically differentiated using the hanging drop technique [47]. Through the use of this method, several differentiated cell types can be obtained, organized in morphologically different areas of a developing clone. We measured different cells in several morphologically distinct regions of the differentiated ES clones. In these clones, three distinct TFIIH mobility groups were observed (Figure 5C and Figure S16). The vast majority of cells (∼70%) presented a high TFIIH mobility, a second group (∼20%) presented an intermediate (∼40%) bound fraction of TFIIH, and a small fraction of cells (∼10%) presented a high TFIIH binding capacity (> than 70%). Postlabeling showed that cells with high TFIIH binding were mainly osteocytes (Figure S17A and S17B). During ES hanging drop differentiation, cardiac myocytes were produced in culture. Morphologically indistinguishable from other cell types, however, cardiac myocytes have the property to beat in vitro, making them easily recognizable, but not easily measurable. Addition of Ca^2+^-free medium impedes cardiac myocytes beating, allowing measuring of TFIIH mobility in these differentiated cells. In these cells, we measured a TFIIH bound fraction of 43%, an intermediate fraction between the mobility observed in cardiac myocytes in situ and the undifferentiated ES cells. All together, our results show that during cellular differentiation of some cell types (neurons, hepatocytes, osteocytes, thricocytes, and myocytes), the dynamic organization of the basal transcription machinery is radically changed, whereas in other cell types (keratinocytes, fibroblasts, and chondrocytes), the dynamic framework of TFIIH activity is maintained.

## Discussion

Previous live-cell studies on complex multifactorial chromatin-associated processes that take place in mammalian cell nuclei, such as transcription, replication, and various DNA repair processes, have disclosed a general model in which highly mobile proteins (process factors) interact with sites of activity (e.g., promoters or DNA lesions) on the basis of stochastic collisions to form transient local machineries in an ordered but highly versatile manner [16],[48],[49]. These biologically relevant novel concepts, however, have been obtained mainly by exogenous expression of tagged factors within highly replicative cells in culture.

In an attempt to study the essential basal transcription initiation factor TFIIH within cells embedded in their natural environment (tissue), we designed a mouse model that allows quantitative determination of TFIIH dynamics. Using carefully designed targeted integration of the live-cell marker YFP at the Xpb locus (expressing the TFIIH subunit XPB), we obtained expression of functional YFP-tagged XPB protein under control of the endogenous promoter (guaranteeing physiological expression); we now find evidence for a fundamentally different scenario for the organization of basic transcription initiation in some cell types in the organism. Postmitotic cells (neurons, myocytes, and hepatocytes, etc.) appear to apply a largely static organization of transcription initiation with components being stably bound to chromatin that otherwise in other cell types (fibroblasts, chondrocytes, and keratinocytes, etc.) exchange constantly in a highly dynamic manner. In postmitotic cells, TFIIH is bound to promoters with a much longer residence time than in proliferative cells. A possible explanation for this static behavior is that in these postmitotic terminally differentiated cells, a large part of the transcription program is dedicated to a specific subset of genes defining cellular specialty and housekeeping functions, without the need to continuously switch to transcribed genes that are involved in proliferation (cell cycle, replication, and mitosis, etc.). It is possible that regular replication of the genome in proliferating (cultured) cells and tissues causes a continuous resetting of the transcription regulation machinery after each round of cell division and, in parallel, would involve a more open and accessible chromatin conformation.

**Figure 6:**
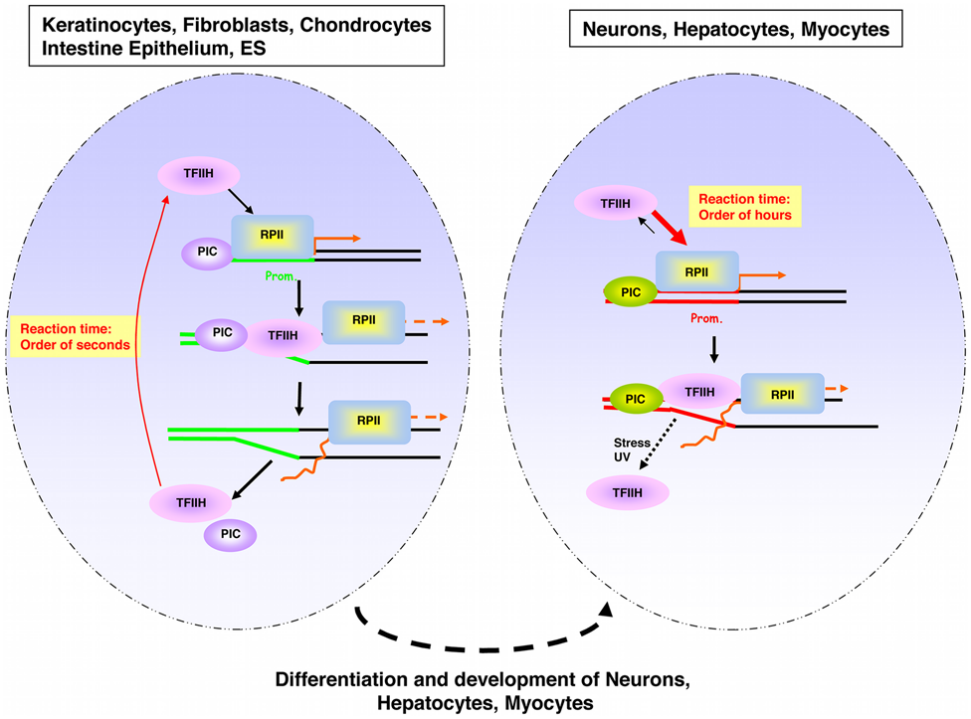
Schematic model of TFIIH switch in mobility during differentiation. Schematic illustration of the nucleus of a proliferative (left) and a postmitotic (right) cell. Green lines (left) and red lines (right) identify the promoter regions. Orange arrows depict the transcriptional start. PIC represents the preinitiation complex. RPBII represents the RNA polymerase II. The arrow width indicates the preferential direction of the equilibrium.

It is generally accepted that differentiation requires and causes a resetting of the transcriptional program by activating and down-regulating specific genes in response to internal and external stimuli, thereby utilizing lineage-specific transcription activators and/or repressors. However, here, we have identified a novel concept of differentiation-dependent spatio-temporal organization of transcription initiation. This concept implies that transcription initiation factors, such as TFIIH, will be bound to promoters much longer in certain cell types than in others (Figure 6). Previously, differences in dynamic associations of lineage-specific transcriptional activators [50],[51] and elongation factors [21],[52] have been linked to activation of transcription. However, the herein described dynamic association of the basal initiation factor TFIIH in neurons, hepatocytes, and myocytes is likely not linked to a higher level of transcription than in, for example, keratinocytes, fibroblasts, or ES cells. We propose a model that the observed low mobility of TFIIH in highly differentiated postmitotic cells is derived form the establishment of a differentiation- and lineage-specific transcriptional program. However, slow mobility of TFIIH is not a general differentiation-dependent phenomenon because in, e.g., fibroblasts, chondrocytes, and keratinocytes, although differentiated, a much higher mobility of TFIIH is present. This observation excludes that this phenomenon is caused by a general change in mobility of histones during ES differentiation, as was previously described [53].

The fast TFIIH remobilization observed in neurons after a cold shock or the induction of local DNA damage demonstrates that, within neurons, TFIIH is still able to respond promptly to a “stress situation” that requires a rapid adaptation of the transcriptional program (in the case of the cold-shock response) or to be implicated in DNA repair. Thus remarkably, the static involvement of TFIIH in transcription initiation does not interfere with the flexibility of cells to change the nuclear organization in response to changing conditions. It is of interest to know how multifunctional factors such as TFIIH are still able to switch from one functional role to another and to relocate to other activity sites despite their virtually immobile nature. Further analysis is required to identify which subroute of NER (global genome NER, transcription-coupled NER, or differentiation-associated repair [DAR] [54]) is employed to repair genomic injuries in differentiated cells or whether lesions in permanently inactive sequences are repaired at all. The strategy outlined here has allowed us to address how transcription is organized in fully differentiated tissues or organs and during differentiation and development. Insights into these processes at the level of an intact organism are also relevant for a better understanding of the molecular basis of cancer and aging-related pathology. Importantly, our mouse model can be crossed into different genetic backgrounds, including existing TFIIH mutated mouse models [12],[55], mutated in another TFIIH subunit, i.e., XPD. These mice are associated with a puzzling clinical heterogeneity ranging from cancer predisposition to dramatically accelerated aging [1],[6]–[8],[12],. For instance, investigating TFIIH engagements directly in affected cells (neurons) in living tissues of XP/CS or TTD mice, which harbor a mutation in one of the other TFIIH components (XPD) [56], will help to elucidate the peculiar phenotype observed in these syndromes.

## Materials and Methods

### Ethics Statement

All animal work have been conducted according to Federation of European Laboratory Animal Science Associations (FELASA) ethical requirements and according to the respect of the 3R animal welfare rules.

### Generation of the Targeting Construct

The knock-in targeting vector (backbone pGEM5ZF) consisted of an approximately 7 Kb (NsiI/SalI fragment) mouse genomic DNA (isogenic to 129 OLA), which contains the 3′ part of the *Xpb* locus. The locus was modified by site-directed mutagenesis (SDM) to transform the stop codon into a coding amino acid (tryptophan) and two unique restriction sites were inserted for cloning purposes (i.e., SacII at the stop codon and NotI at a distance of 20 bp downstream of the SacII site). Between the SacII and NotI, a modified YFP was cloned, the YFP start was modified into a valine to avoid undesired translation of nonfused YFP. The YFP gene was further tagged at the C-terminus with a stretch of six histidines and an HA epitope sequence. A unique ClaI site was created 10 bp downstream of the stop codon of the modified YFP to introduce a neomycin gene-expression cassette, flanked by two LoxP sites [57], used as a dominant selectable marker. The dominant marker was inserted in the same transcriptional orientation as the *Xpb* gene.

### ES Cell Culture and Gene Targeting

ES cells (129 Ola, subclone IB10) were cultured in BRL-conditioned medium supplemented with 1,000 U/ml leukemia inhibitory factor. A total of 20 µg of the PmeI linearized targeting vector was electroporated into approximately 10^7^ ES cells in 500 µl. Selection with 0.2 µg/ml G418 was started 24 h after electroporation. After 8–10 d, G418 resistant clones were isolated. Screening for homologous recombinants was performed using DNA blot analyses of NcoI-digested DNA with a 400-bp 5′ external probe (see Figure 1A and Figure S1A). Out of 128 G418 resistant clones, 12 ES clones had a correctly targeted *Xpb* allele. Two out of the 12 correctly targeted ES clones, checked for proper caryotype, were injected into blastocysts of C57Bl/6 mice and transplanted into B10/CBA foster mothers. Chimeric mice were further crossed, and germline transmission of the targeted allele to offspring was genotyped by PCR using primer sets (as described in Figure S1) and genotyping of offspring was done by PCR (see Figure S1). Primers sequences are available on request.

### Mice

The knock-in *Xpb^y^* (resulting fusion gene between *Xpb* and YFP, coding for XPB-YFP) allele was maintained in both FVB and C57BL/6 backgrounds. We thank P. Vassalli for pCAGGSCre plasmid used to generate the transgenic CAG-Cre recombinase–expressing mice. EGFP-expressing mice were kindly provided by Dr. Okabe [33] and Dr. R. Torensma (Nijmegen).

### Cell Culture and Specific Treatments

Murine dermal fibroblasts (MDF) were extracted from *Xpb^+/+^*, *Xpb^y/y^*, and *Xpb^y/y^*•*Xpc^−/−^* mice, following established procedures [58] and cultured in a 1∶1 mixture of Ham's F10 and DMEM (Gibco) supplemented with antibiotics and 10% fetal calf serum at 37°C, 3% O_2_, and 5% CO_2_. To induce in vitro differentiation of XPB-YFP–expressing ES cells, we applied the hanging drop method [47]. Briefly, 20 µl of ES cell suspension (2×10^5^/ml in DMEM [Lonza] with 20% fetal calf serum [Lonza], 50 U penicillin/ml, and 50 µg streptomycin/ml [Lonza], 1% nonessential amino acids [Lonza], 0.1 mM B-mercaptoethanol [Sigma]) were placed on the lids of Petri dishes filled with PBS. After culturing for 3 d, the aggregates were transferred into bacteriological Petri dishes. Two days later, embryoid bodies were placed in Costar six-well plates with gelatin-coated coverslips for further development into different cell tissues. From day 3 on, retinoic acid (10^−8^ M) was added to induce skeletal muscle differentiation. Treatment with ultraviolet (UV) light at 254 nm (UV-C) was performed using a Philips germicidal lamp. For UV-survival experiments, cells were exposed to different UV-C doses, 2 d after plating. Survival was determined 3 d after UV irradiation by incubation at 37°C with ^3^H-thymidine, as described previously [59]. For unscheduled DNA synthesis (UDS), cells were exposed to 8 J/m^2^ of UV-C and processed as described previously [28].

### Organotypic Cultures

Organotypic explants of cerebral cortex and cerebellum were produced as previously described [31],[60]. Tissues were analyzed on the same day of extraction in Neurobasal-A (GIBCO) medium, supplemented with antibiotics and B-27 at 37°C, 3% O_2_, and 5% CO_2_. FRAP analysis on cells within organotypic slices, maintained in culture for 1 wk, gave the same results as when performed on freshly extracted slices. Organotypic slices of heart, liver, and intestine were produced by cutting 300 µm of the organ with a Tissue Chopper (McIllwain). Slices were analyzed within 2 h following preparation, unless differently specified in the text. Skin tissues (epidermis and dermis) were prepared as described [10]. Before imaging, the two layers were mechanically separated and mounted on a coverslip for imaging and analysis.

### Immunoblot Analysis

Whole-cell extracts (WCE) of *Xpb^+/+^* and *Xpb^y/+^* ES cells were prepared by isolating cells from a semiconfluent Petri dish (10 cm). Cells were washed with phosphate-buffered saline (PBS) and homogenized by sonication. Western blot analysis was performed as previously described [1].

### Comparative Immunofluorescence Assays

A mixture of Xpb^+/+^ and Xpb^y/y^ cells were grown on glass coverslips and fixed with 2% paraformaldehyde at 37°C for 15 min. Immunofluorescence analysis was carried out as previously described [1]. Antibodies use were a rabbit polyclonal anti XPB (1∶500, S-19, Santa Cruz Biotechnology), a mouse monoclonal anti-p62 (1∶1,000, 3C9, kindly provided by Dr. J. M. Egly), and a rat monoclonal anti-HA (1∶1,000, 3F10, Roche).

### Fluorescence Recovery after Photobleaching (FRAP) and Calculation of TFIIH Bound Fractions

FRAP experiments were performed as described before [24],[61] at high time resolution on a Zeiss LSM 510 meta confocal laser scanning microscope (Zeiss). Briefly, a narrow strip spanning the nucleus of a cell was monitored every 200 ms at 1% laser intensity (30 mW argon laser, current set at 6.5 A, 514-nm line) until the fluorescence signal reached a steady level (after circa 4 s). The same strip was then photobleached for 60 ms at the maximum laser intensity. Recovery of fluorescence in the strip was then monitored every 200 ms for about 30 s (1% laser intensity). All FRAP data was normalized to the average prebleached fluorescence after removal of the background signal. Every plotted FRAP curve is an average of at least ten measured cells.

To estimate the relative TFIIH bound fractions (BF) from the FRAP measurements, we used the first data point after photobleaching (*F*_min_) as an approximation of the baseline fluorescence recovery (BF = 1), i.e., the fluorescence level in the absence of recovery, when all proteins are considered immobile. We calculated the time-average fluorescence signal taken between 2 and 3.9 s prior to the photobleaching step to obtain the average prebleach fluorescence (*F*_pre_), and then between 10 and 15 s to estimate the final fluorescence recovery level (*F*_max_). The bound fraction is then given by:
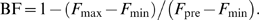



We corrected for the photobleached fraction, i.e., the incomplete recovery of fluorescence due to irreversible YFP bleaching during the FRAP procedure, as follows: whole nuclei of cultured keratinocytes were first imaged, subsequently strip-bleached (60-ms photobleach at maximum laser intensity), then imaged immediately after the bleach pulse and again 1 min later when no traces of the bleached strip were observed. The photobleached fraction (PBF) relative to the baseline fluorescence recovery (*F*_min_) was estimated as the average fluorescence intensity loss between the prebleached image (*F*_nucleus_ (pre)) and the last image of the nucleus (*F*_nucleus_ (last)):
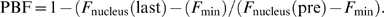
The corrected bound fraction is then given by:
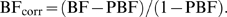



Note: Measuring conditions where designed solely to measure immobile fractions, not diffusion or dissociation constants.

### Multiphoton Laser-Induced DNA Damage

Laser-induced DNA damage was conducted as previously described [45]. Briefly, a Coherent Verdi pump laser with a Mira 900 mode locked Ti:Sapphire laser system (Coherent) was directly coupled to a LSM 510 NLO microscope (Zeiss) to obtain an 800-nm pulsed output (200-fs pulse width at 76 MHz, 10 mW output at the sample). Single nuclei targeted with the multiphoton laser received an approximately 250-ms exposure restricted to a 1-µm-wide strip.

### Chromatin Immunoprecipitation (ChIP)

The applied ChIP protocol was adapted from previously described methods [62],[63]. Briefly, brain fragments were fixed by adding 11% formaldehyde solution containing 50 mM Hepes (pH 8), 1 mM EDTA, 0.5 mM EGTA, and 0.1 M NaCl to a final concentration of 1% and incubated for 15 min at room temperature and 1 h at 4°C. Cross-linking was stopped by addition of glycine to a final concentration of 0.125 M. Fragments were washed twice with cold phosphate-buffered saline and treated with lysis buffer (50 mM Hepes [pH 8], 140 mM NaCl, 1 mM EDTA, 0.5 mM EGTA, 10% glycerol, 0.5% NP-40, 0.25% Triton X-100) containing 1 mM PMSF and a mixture of protease inhibitors. Fragments were homogenized (Ultra turrax, T25 basic), and ground on ice with an A-type and B-type glass pestle (20 strokes each) to allow nuclei release. Nuclear suspension was then sheared extensively by sonication on ice to obtain fragments of 200 to 600 bp (as revealed by ethidium bromide staining of aliquots run on agarose gels). For each ChIP reaction, 100 µg of cross-linked chromatin was immunoprecipitated with 0.5 µg of HA-antibody in RIPA buffer (10 mM Tris-HCl [pH 8], 1 mM EDTA, 0.5 mM EGTA, 140 mM NaCl, 1%Triton X-100, 0.1% Na-deoxycholate, 0.1% sodium dodecyl, 1 mM PMSF, and a mixture of protease inhibitors) overnight. The immunocomplexes were collected by adsorption (3 h) to precleared protein G sepharose beads (Upstate) precoated in RIPA containing 0.1 mg/ml sonicated salmon sperm DNA (ssDNA). The beads were then washed twice with 20 volumes of RIPA and once with RIPA containing ssDNA (Sigma), and twice with RIPA containing ssDNA and 0.3 M NaCl. Finally, the beads were washed with 20 volumes of LiCl buffer (10 mM Tris-HCl [pH 8], 1 mM EDTA, 0.5 mM EGTA, 0.25 M LiCl, 0.5% triton X-100, 0.5% Na-deoxycholate, 1 mM PMSF, and a mixture of protease inhibitors), resuspended in RIPA buffer, and divided into two equal parts to analyze coprecipitating proteins and DNA sequences.

### DNA Extraction after ChIP and PCR Reaction

For DNA analysis, the immunocomplexes were treated with RNAse (5 µg/µl) for 30 min at 37°C and by proteinase K (200 µg/µl) 3 h at 55°C in 50 mM Tris-HCl (pH 8), 1 mM EDTA, 100 mM NaCl, 0.5% SDS. Formaldehyde cross-links were reverted by heating the samples at 65°C for 6 h. The cross-linked DNA was extracted with phenol:chloroform and precipitated with ethanol in the presence of carrier glycogen. Pellets were resuspended in 30 µl of distillated water. Chromatin-immunoprecipitated DNA was subjected to PCR amplification. PCR was performed using 200 ng (for promoter XPB gene amplification) and 100 ng (for untranscribed sequences) of the chromatin immunoprecipitate and 400 nM concentration of both sense and antisense primers (primer sequence available upon request) in a final volume of 25 µl using the PureTaq Ready-to-go PCR beads (GE Healthcare). PCR products were analyzed on 2% agarose7 gels by SYBR Green reagent. Data were analyzed and quantified using Quantity One program (Bio-Rad). For adjustment of amplification efficiency of each primer set, PCR signal intensities from chromatin-immunoprecipitated DNA were normalized to those from the input genomic DNA and expressed as a percentage of the input (gDNA).

### RNA Extraction and Reverse Transcriptase PCR Reaction

Brain slices and cultured cells were incubated at 37°C and 4°C. Additionally, cultured cells were incubated with 0.1 µg/ml of actinomycin D for 2 h. Samples were homogenized in Trizol using the Tissuelyser (Qiagen) for 90 s. RNA isolation was performed using the RNeasy Mini Kit (Qiagen). cDNA was produced using 2 µg of RNA with a Reverse Transcription Kit (Invitrogen), random primers (Invitrogen), 1 µg of cDNA, and 400 nM concentration of both sense and antisense primers (primer sequence and PCR cycle available upon request) in a final volume of 25 µl using the PureTaq Ready-to-go PCR beads (GE Healthcare). PCR products were analyzed on 2.5% agarose gels.

## Supporting Information

Figure S1 **Analysis of the *Xpb* gene targeting.** (A) Southern blot analysis of genomic DNA (NcoI digest) from IB10 ES cells (OLA 129) electroporated with the targeting construct (Figure 1A and Materials and Methods) to identify clones with proper gene targeting by homologous recombination, using a part of exon 14 (*Xpb*) as an external probe (not included in the targeting construct), indicated as a yellow bar. The genomic organization, including relevant restriction sites of both the wild-type (wt) allele and the homologous targeted allele were indicated: exons in orange, YFP coding sequence in green, LoxP_Neo_LoxP cassette in purple, and sizes of NcoI restriction fragments in red. Untargeted genomic DNA from IB10 ES cells (lane 1) and chromosomal DNA of tails from 129 mouse strain (lane 2) were loaded as control. Twelve homologous recombinant ES cell lines (e.g., in lane 3) out of 128 analyzed G418-resistant transformants were obtained (9% targeting frequency). (B) Immunoblot of total cellular protein extracts from three targeted ES clones (lanes 2–4) and a nontargeted ES clone (lane 1) as negative control was performed using an antibody against the HA tag (3F10, Roche). (C) Scheme of the amplification strategy to identify wt, heterozygous, and homozygous animals. Two sets of primers: set A (*I*_forward_ [*I_f_*] + *I*_reverse_ [*I_r_*]) and set B (*I*_forward_ [*I_f_*] + II_reverse_ [II*_r_*]) amplify the untargeted allele (set A) or the targeted allele (set A and B). The size of the fragment amplified by primer set A discriminates the untargeted allele (*Xpb^+^* allele: 470 bp amplification, upper scheme) from the targeted allele containing the neomycin-resistant marker (*Xpb^y−Neo^* allele: >2,500 bp amplification, middle scheme), and from the targeted allele without the NeoMarker (*Xpb^y^* allele: 1,180 bp amplification, lower scheme). (D) Amplification of genomic DNA from untargeted *Xpb^+/+^* (lane 1), heterozygous *Xpb^y/+^* (lane 2), and homozygous *Xpb^y/y^* mice (lane 3) after recombining out the LoxP_Neo_LoxP cassette. Lane M: marker. (E) Amplification of genomic DNA from *Xpb^+/+^* (lane 1and 2), *Xpb^y−Neo/+^* (lane 3 and 4), and *Xpb^y/+^* mice (lane 5 and 6). Lane M: marker.
(0.53 MB TIF)

Figure S2 **Characterization of cell expressing fluorescently tagged TFIIH (XPB-YFP).** (A) Comparative immunofluorescence of a mixed population of dermal fibroblasts isolated from an untargeted mouse (*Xpb^+/+^*, HA negative) and a targeted mouse (*Xpb^y/y^*, HA positive), stained with anti-p62 (red, left) or anti-HA (green, right), which recognizes the XPB-YFP-His_6_HA protein. Bar: 10 µm. (B) DNA repair or unscheduled DNA synthesis (UDS); expressed as percentage of wild-type (wt) UDS (NER-proficient cells assayed in parallel) of dermal fibroblasts isolated from *Xpb^+/+^* mice (wt, green), *Xpb^y/y^* mice (red), and *Xpb^y/y^* mice crossed with *Xpc^−/−^* (NER-deficient) mice (blue). UDS in *Xpb^+/+^* was set at 100%. (C) Cultured cells derived from the Xpb^y/y^ mouse model. The YFP signal and transmission image were merged from a primary cultures of dermal fibroblasts (left panel) and keratinocytes (right panel). Note the uniform expression of TFIIH in both cell types. Bar: 20 µm.
(1.55 MB DOC)

Figure S3 **TFIIH expression and mobility in neurons.** (A) Image of an organotypic tissue slice of cortex brain from Xpb^y/y^. Bar: 50 µm. (B) Prolonged time-lapse imaging of a cortex neuron after strip-FRAP. Time scale is expressed in minutes.
(3.17 MB DOC)

Figure S4 **Strip-FRAP graphs used to calculate TFIIH bound fractions indicated in** **Figure 2C** **.** The dotted square indicates the approximate time frame used to calculate the immobile fractions. Mean SEM is the average standard error of the mean calculated over the entire time range of each FRAP curve.
(0.25 MB TIF)

Figure S5 **Mobility of TFIIH and free-GFP in neurons from the cerebellum.** (A) TFIIH mobility in Purkinje cells and granular neurons in cerebellar organotypic slices. Left upper panel: immunohistochemistry of a cerebellum paraffin-embedded section stained with an HA antibody (brown). Left bottom panel: image of DAPI-stained Purkinje cell and a granular neuron, bar: 10 µm. Right panel: FRAP curve of Purkinje cells (red) and granular neurons (blue), showing the same TFIIH in these two different neuron types having different TFIIH concentrations and chromatin make-ups. (B) Mobility of free GFP in granular neurons (GN) of the cerebellum from mice that ubiquitously express nontagged GFP. Left panel: image of GFP-expressing GN, the dotted circle indicates the nuclear contour, bar: 5 µm. Right panel: FRAP curve of free GFP (red), indicating that nonfused GFP is freely mobile in GN.
(1.01 MB TIF)

Figure S6 **Strip-FRAP graphs used to calculate TFIIH bound fractions indicated in** **Figure 3A** **, section DNA repair.** In blue is TFIIH mobility from cortex neurons of XPBYFP mice and in red, TFIIH mobility from cortex neurons of XPBYFP mice crossed into the XPC background. The dotted square indicates the approximate time frame used to calculate the immobile fractions. Mean SEM is the average standard error of the mean calculated over the entire time range of each FRAP curve.
(0.19 MB TIF)

Figure S7 **Strip-FRAP graphs used to calculate TFIIH bound fractions indicated in** **Figure 3A** **, section Pol 2 transcription.** The dotted square indicates the approximate time frame used to calculate the immobile fractions. Mean SEM is the average standard error of the mean calculated over the entire time range of each FRAP curve.
(0.26 MB TIF)

Figure S8 **α-Amanitin transcription inhibition.** (A) Confocal imaging of chondrocytes isolated from *Xpb^y/y^* mice, untreated (left panel) and (B) treated (right panel) with α-amanitin. Transcription activity has been measured by incorporating BrU into nascent m-RNA. Anti-BrU (red), YFP (green), and Dapi (Blue).
(1.60 MB DOC)

Figure S9 **RNAP1 and RNAP2 transcription in neurons.** (A) Image of a cortex neuron within organotypic slice in transmitted light (TL) (left panel) and fluorescent light (middle panel). Bar: 10 µm. Left panel: image shows a cortex neuron within an organotypic brain slice 10 min after bleaching part of the nucleolus. (B) Semiquantitative reverse transcriptase (RT)-PCR of pre-rRNA extracted from organotypic cortex slices and cultured chondrocytes (left panel) and Hela cells (right panel), incubated at different temperatures and treated with actinomycin D. (C) Nuclear extract from the brain was immunoprecipitated with HA-antibodies and subjected to Western blot analysis with the indicated antibodies. The input lanes represent Immunoprecipitation fraction (IP), cleared chromatin (CC) cross-linked extract before IP, supernatant (S) of cross-linked extract after IP. (D) Bar graphs representing the quantification of a semiquantitative PCR on 200 ng of genomic DNA (gDNA) and 200 ng of HA-precipitated chromatin from cortex slice incubated at 37°C and 4°C. Promoter sequence from Pol1 gene (−119/+7) and untranscribed region adjacent to the Pol1 gene (Chr 6: 71937600–71938000). The *y*-axis represents the percentage of amplified sequences from the HA-precipitated chromatin versus amplified sequences from gDNA, set to 100%. Error bars have been calculated for three biological replicates.
(0.94 MB DOC)

Figure S10 **Strip-FRAP graphs used to calculate TFIIH bound fractions indicated in** **Figure 3A** **, section Pol 1 transcription.** The dotted square indicates the approximate time frame used to calculate the immobile fractions. Mean SEM is the average standard error of the mean calculated over the entire time range of each FRAP curve.
(0.23 MB TIF)

Figure S11 **Strip-FRAP graphs used to calculate TFIIH bound fractions indicated in** **Figure 4A** **.** The dotted square indicates the approximate time frame used to calculate the immobile fractions. Mean SEM is the average standard error of the mean calculated over the entire time range of each FRAP curve.
(0.29 MB TIF)

Figure S12 **TFIIH mobility in chondrocytes.** (A) Confocal imaging of chondrocytes isolated from *Xpb^y/y^* mice proliferating (left panels) and confluent (right panels). Ki67 immunostaining (in red) was used to confirm the proliferative state of the cells. (B) TFIIH mobility in proliferating (blue) and confluent (red) chondrocytes.
(1.34 MB TIF)

Figure S13 **Strip-FRAP graphs used to calculate TFIIH bound fractions indicated in** **Figure 4B** **.** The dotted square indicates the approximate time frame used to calculate the immobile fractions. Mean SEM is the average standard error of the mean calculated over the entire time range of each FRAP curve.
(0.27 MB TIF)

Figure S14 **Strip-FRAP graphs used to calculate TFIIH bound fractions indicated in** **Figure 4C** **.** The dotted square indicates the approximate time frame used to calculate the immobile fractions. Mean SEM is the average standard error of the mean calculated over the entire time range of each FRAP curve.
(0.29 MB TIF)

Figure S15 **Strip-FRAP graphs used to calculate TFIIH bound fractions indicated in** **Figure 5B** **.** The dotted square indicates the approximate time frame used to calculate the immobile fractions. Mean SEM is the average standard error of the mean calculated over the entire time range of each FRAP curve.
(0.24 MB TIF)

Figure S16 **Strip-FRAP graphs used to calculate TFIIH bound fractions indicated in** **Figure 5C** **.** The dotted square indicates the approximate time frame used to calculate the immobile fractions. Mean SEM is the average standard error of the mean calculated over the entire time range of each FRAP curve.
(0.29 MB TIF)

Figure S17 **Differentiation markers in ES clones.** (A) Transmission light image of a differentiated ES clone stained with Alizarin Red. Red-stained cells and inclusion are characteristically highly mineralized (Ca^2+^-containing) cells. (B) Transmission light image of a differentiated ES clone stained with alkaline phosphatase. Violet-stained cells are osteocytes producing the enzyme.
(5.88 MB TIF)

## Abbreviations

CS: Cockayne syndrome
ES: embryonic stem
FRAP: fluorescence recovery after photobleaching
NER: nucleotide excision repair
TTD: trichothiodistrophy
XP: xeroderma pigmentosum
