# Supplementary figures and images for: Differentiation Driven Changes in the Dynamic Organization of Basal Transcription Initiation
Source: PLoS Biol. 2009 Oct 20;7(10):e1000220. doi: 10.1371/journal.pbio.1000220 (PMC2754661; doi:10.1371/journal.pbio.1000220)

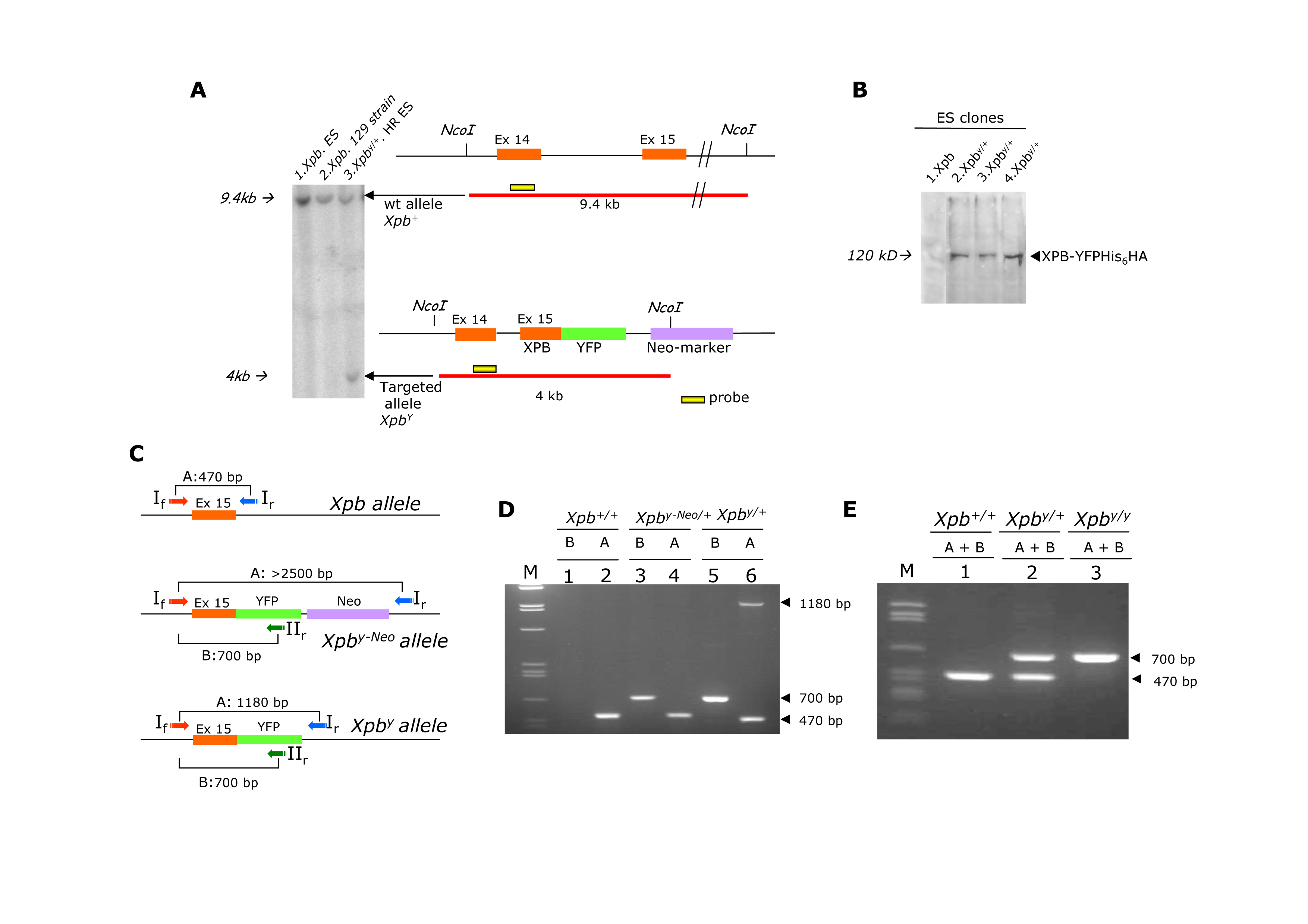

Supplement: Figure S1 — Analysis of the Xpb gene targeting. (A) Southern blot analysis of genomic DNA (NcoI digest) from IB10 ES cells (OLA 129) electroporated with the targeting construct (Figure 1A and Materials and Methods) to identify clones with proper gene targeting by homologous recombination, using a part of exon 14 (Xpb) as an external probe (not included in the targeting construct), indicated as a yellow bar. The genomic organization, including relevant restriction sites of both the wild-type (wt) allele and the homologous targeted allele were indicated: exons in orange, YFP coding sequence in green, LoxP_Neo_LoxP cassette in purple, and sizes of NcoI restriction fragments in red. Untargeted genomic DNA from IB10 ES cells (lane 1) and chromosomal DNA of tails from 129 mouse strain (lane 2) were loaded as control. Twelve homologous recombinant ES cell lines (e.g., in lane 3) out of 128 analyzed G418-resistant transformants were obtained (9% targeting frequency). (B) Immunoblot of total cellular protein extracts from three targeted ES clones (lanes 2–4) and a nontargeted ES clone (lane 1) as negative control was performed using an antibody against the HA tag (3F10, Roche). (C) Scheme of the amplification strategy to identify wt, heterozygous, and homozygous animals. Two sets of primers: set A (I forward [If] + I reverse [Ir]) and set B (I forward [If] + IIreverse [IIr]) amplify the untargeted allele (set A) or the targeted allele (set A and B). The size of the fragment amplified by primer set A discriminates the untargeted allele (Xpb+ allele: 470 bp amplification, upper scheme) from the targeted allele containing the neomycin-resistant marker (Xpby−Neo allele: >2,500 bp amplification, middle scheme), and from the targeted allele without the NeoMarker (Xpby allele: 1,180 bp amplification, lower scheme). (D) Amplification of genomic DNA from untargeted Xpb+/+ (lane 1), heterozygous Xpby/+ (lane 2), and homozygous Xpby/y mice (lane 3) after recombining out the LoxP_Neo_LoxP cassette. [file pbio.1000220.s001.tif]

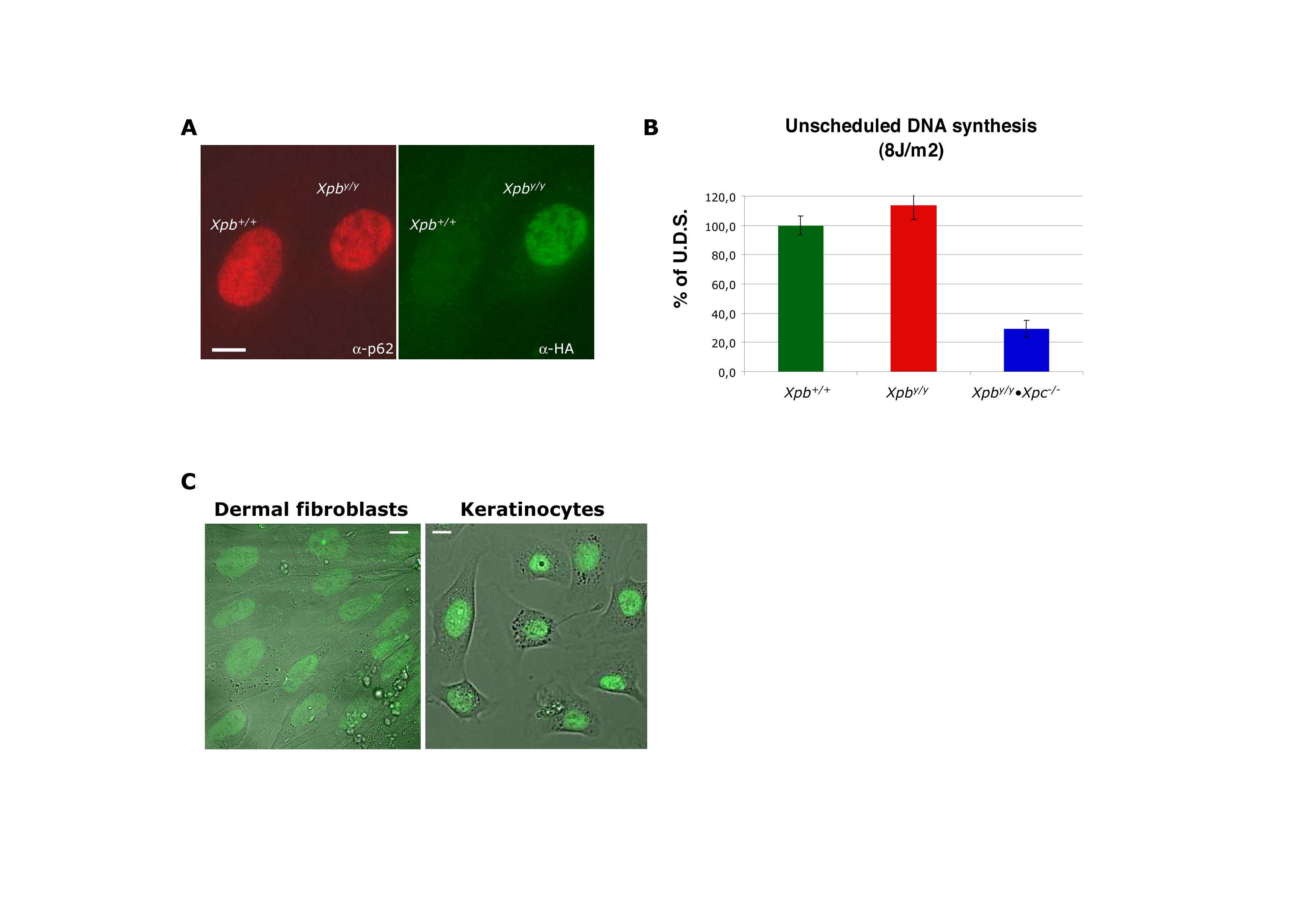

Supplement: Figure S2 — Characterization of cell expressing fluorescently tagged TFIIH (XPB-YFP). (A) Comparative immunofluorescence of a mixed population of dermal fibroblasts isolated from an untargeted mouse (Xpb+/+, HA negative) and a targeted mouse (Xpby/y, HA positive), stained with anti-p62 (red, left) or anti-HA (green, right), which recognizes the XPB-YFP-His6HA protein. Bar: 10 µm. (B) DNA repair or unscheduled DNA synthesis (UDS); expressed as percentage of wild-type (wt) UDS (NER-proficient cells assayed in parallel) of dermal fibroblasts isolated from Xpb+/+ mice (wt, green), Xpby/y mice (red), and Xpby/y mice crossed with Xpc−/− (NER-deficient) mice (blue). UDS in Xpb+/+ was set at 100%. (C) Cultured cells derived from the Xpby/y mouse model. The YFP signal and transmission image were merged from a primary cultures of dermal fibroblasts (left panel) and keratinocytes (right panel). Note the uniform expression of TFIIH in both cell types. Bar: 20 µm. (1.55 MB DOC) [file pbio.1000220.s002.tif]

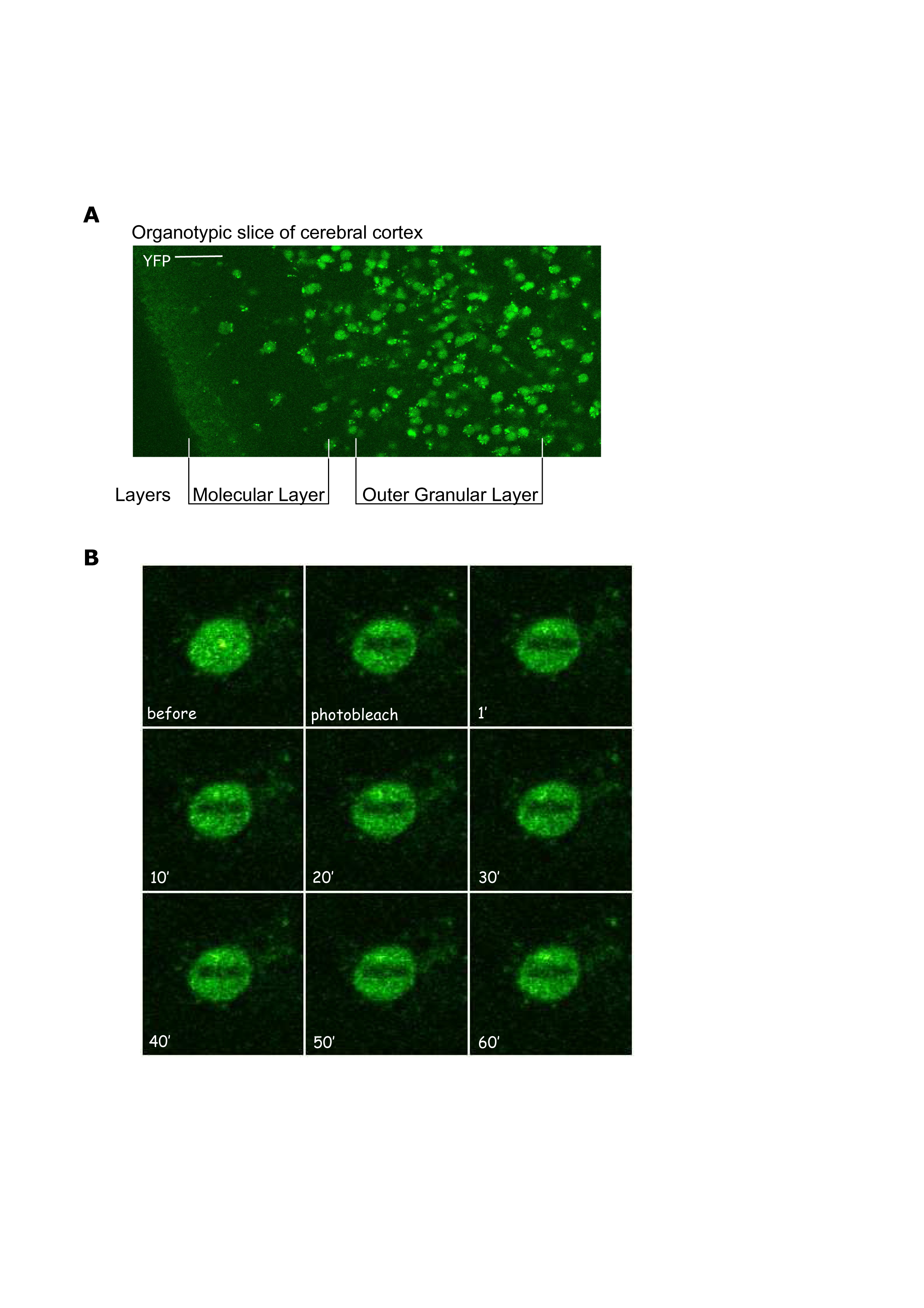

Supplement: Figure S3 — TFIIH expression and mobility in neurons. (A) Image of an organotypic tissue slice of cortex brain from Xpby/y. Bar: 50 µm. (B) Prolonged time-lapse imaging of a cortex neuron after strip-FRAP. Time scale is expressed in minutes. (3.17 MB DOC) [file pbio.1000220.s003.tif]

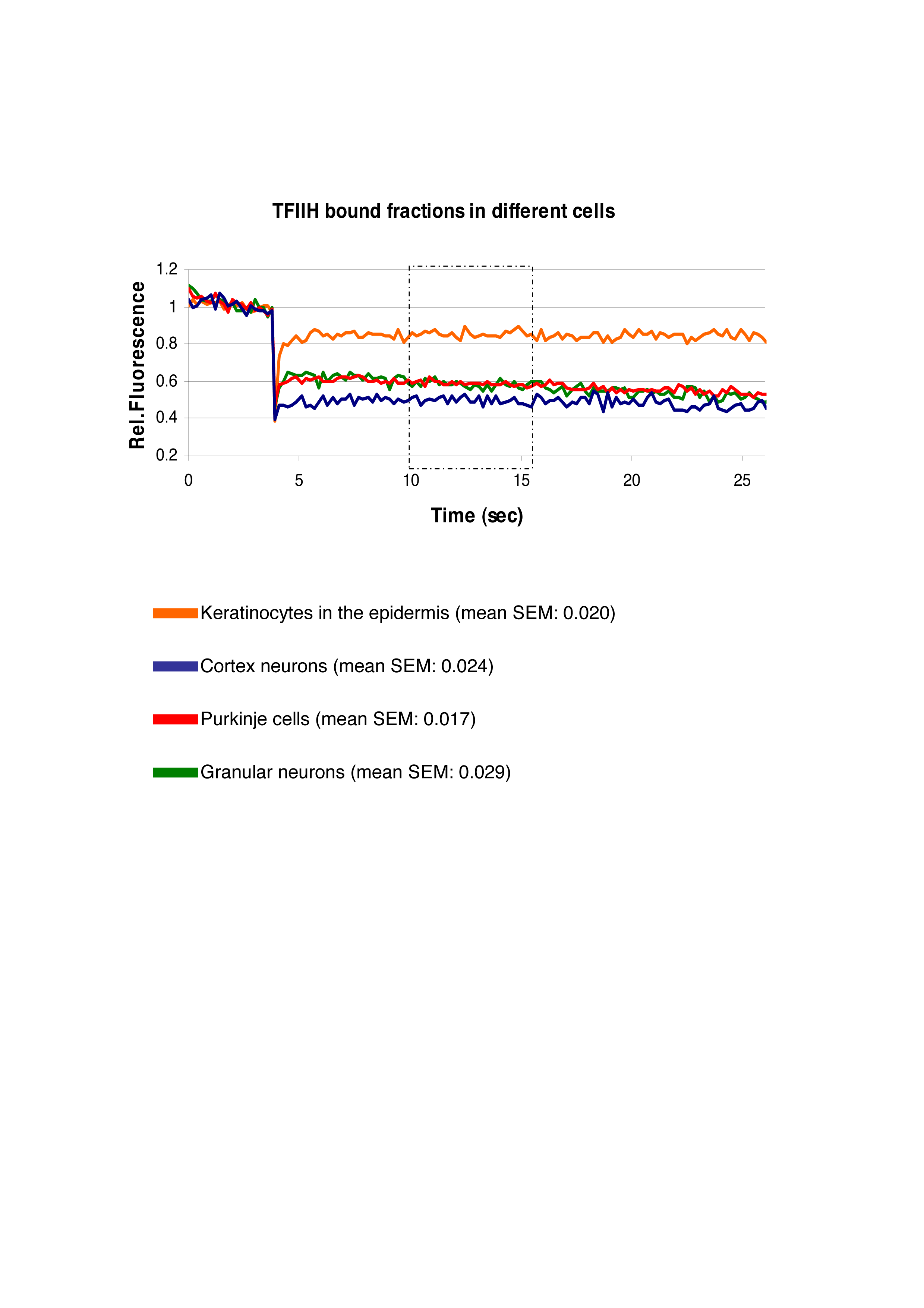

Supplement: Figure S4 — Strip-FRAP graphs used to calculate TFIIH bound fractions indicated in Figure 2C . The dotted square indicates the approximate time frame used to calculate the immobile fractions. Mean SEM is the average standard error of the mean calculated over the entire time range of each FRAP curve. (0.25 MB TIF) [file pbio.1000220.s004.tif]

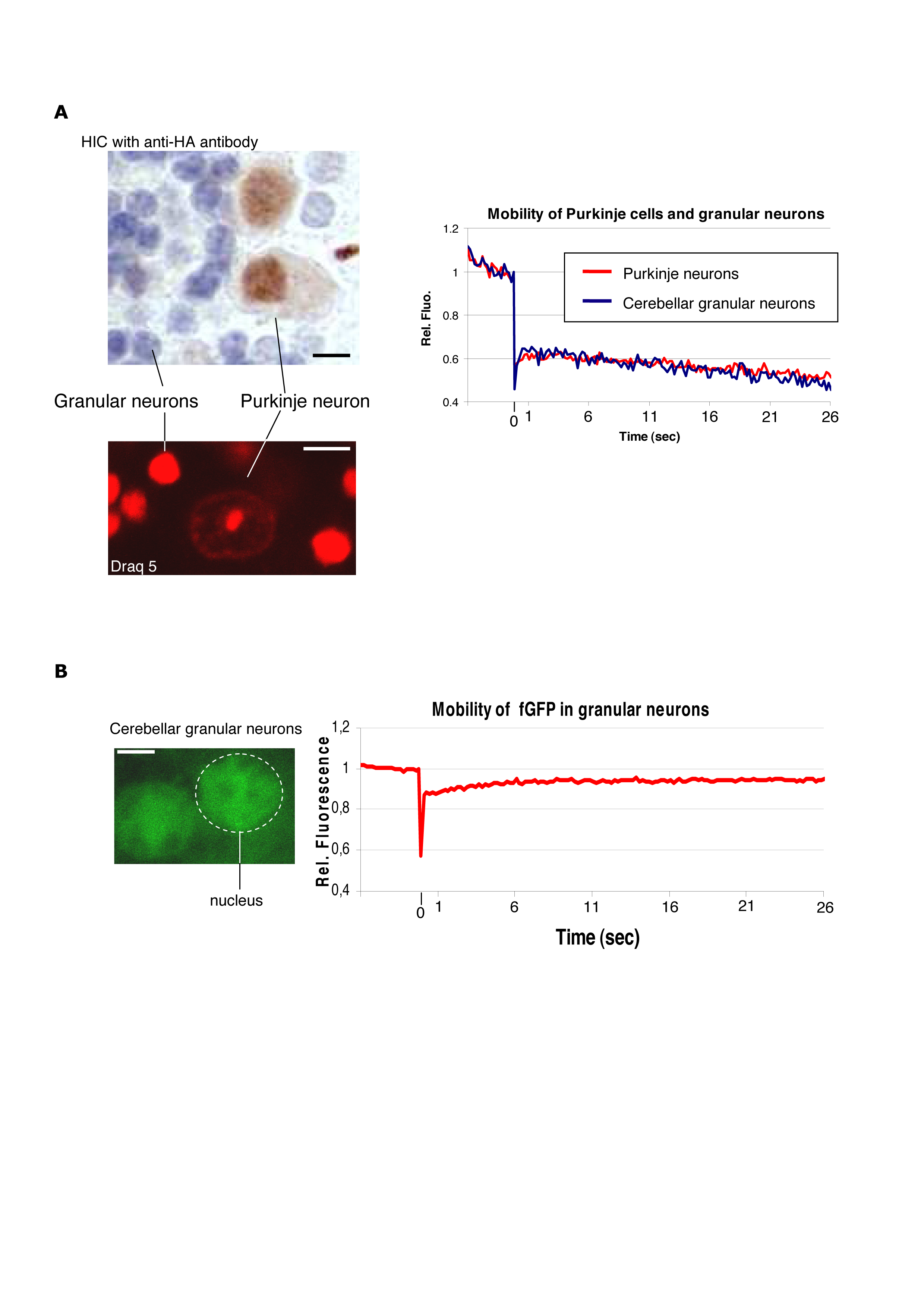

Supplement: Figure S5 — Mobility of TFIIH and free-GFP in neurons from the cerebellum. (A) TFIIH mobility in Purkinje cells and granular neurons in cerebellar organotypic slices. Left upper panel: immunohistochemistry of a cerebellum paraffin-embedded section stained with an HA antibody (brown). Left bottom panel: image of DAPI-stained Purkinje cell and a granular neuron, bar: 10 µm. Right panel: FRAP curve of Purkinje cells (red) and granular neurons (blue), showing the same TFIIH in these two different neuron types having different TFIIH concentrations and chromatin make-ups. (B) Mobility of free GFP in granular neurons (GN) of the cerebellum from mice that ubiquitously express nontagged GFP. Left panel: image of GFP-expressing GN, the dotted circle indicates the nuclear contour, bar: 5 µm. Right panel: FRAP curve of free GFP (red), indicating that nonfused GFP is freely mobile in GN. (1.01 MB TIF) [file pbio.1000220.s005.tif]

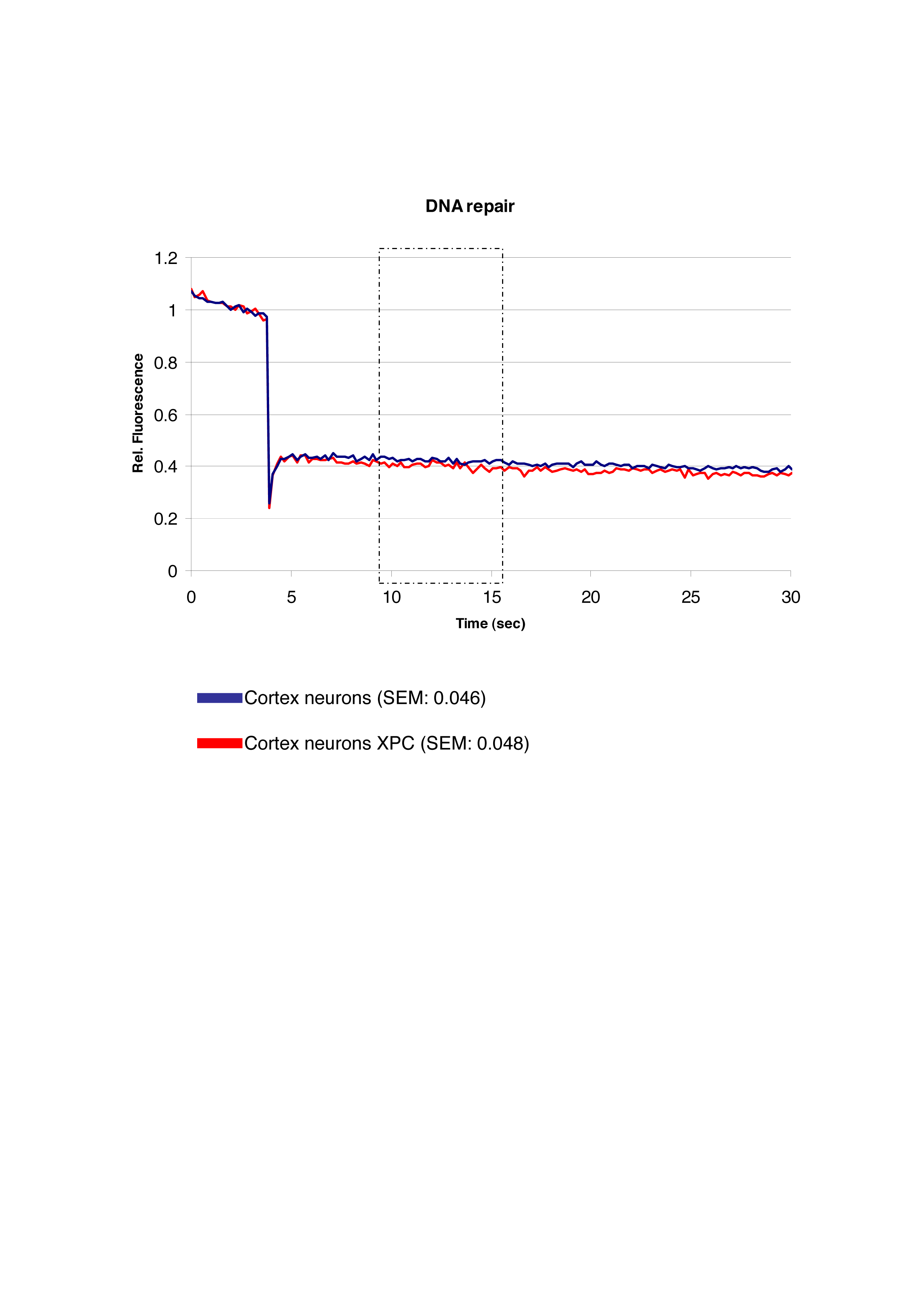

Supplement: Figure S6 — Strip-FRAP graphs used to calculate TFIIH bound fractions indicated in Figure 3A , section DNA repair. In blue is TFIIH mobility from cortex neurons of XPBYFP mice and in red, TFIIH mobility from cortex neurons of XPBYFP mice crossed into the XPC background. The dotted square indicates the approximate time frame used to calculate the immobile fractions. Mean SEM is the average standard error of the mean calculated over the entire time range of each FRAP curve. (0.19 MB TIF) [file pbio.1000220.s006.tif]

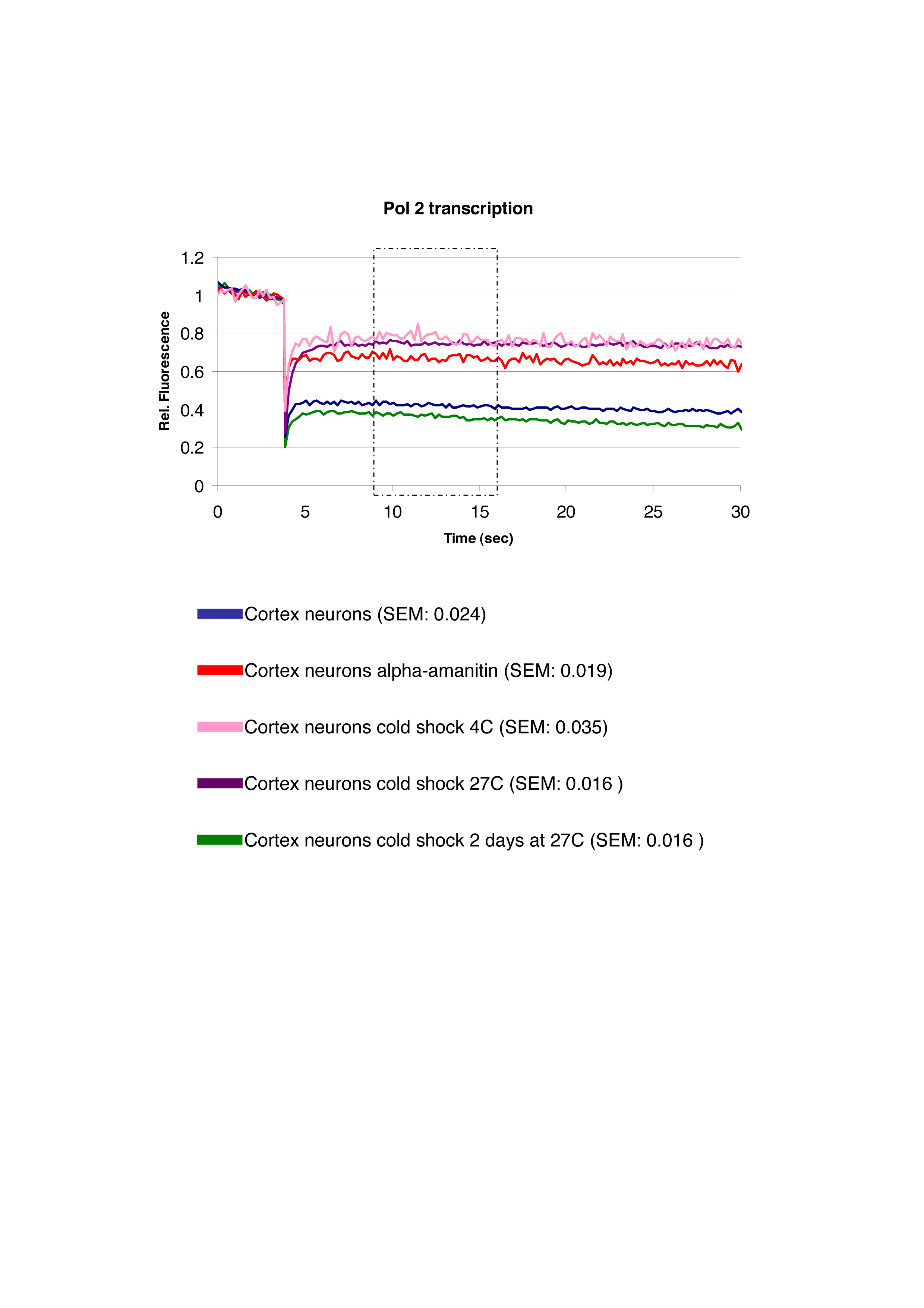

Supplement: Figure S7 — Strip-FRAP graphs used to calculate TFIIH bound fractions indicated in Figure 3A , section Pol 2 transcription. The dotted square indicates the approximate time frame used to calculate the immobile fractions. Mean SEM is the average standard error of the mean calculated over the entire time range of each FRAP curve. (0.26 MB TIF) [file pbio.1000220.s007.tif]

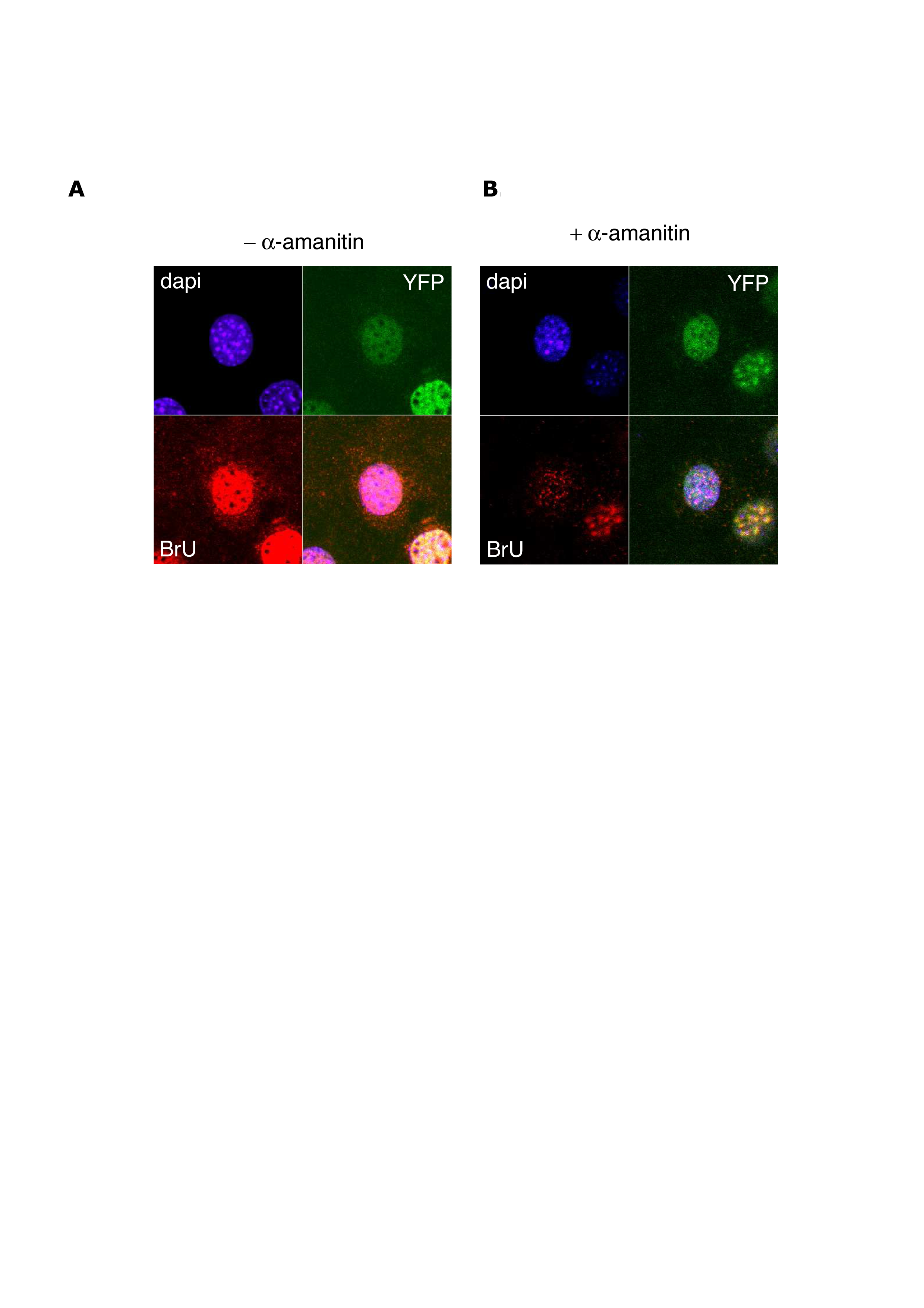

Supplement: Figure S8 — α-Amanitin transcription inhibition. (A) Confocal imaging of chondrocytes isolated from Xpby/y mice, untreated (left panel) and (B) treated (right panel) with α-amanitin. Transcription activity has been measured by incorporating BrU into nascent m-RNA. Anti-BrU (red), YFP (green), and Dapi (Blue). (1.60 MB DOC) [file pbio.1000220.s008.tif]

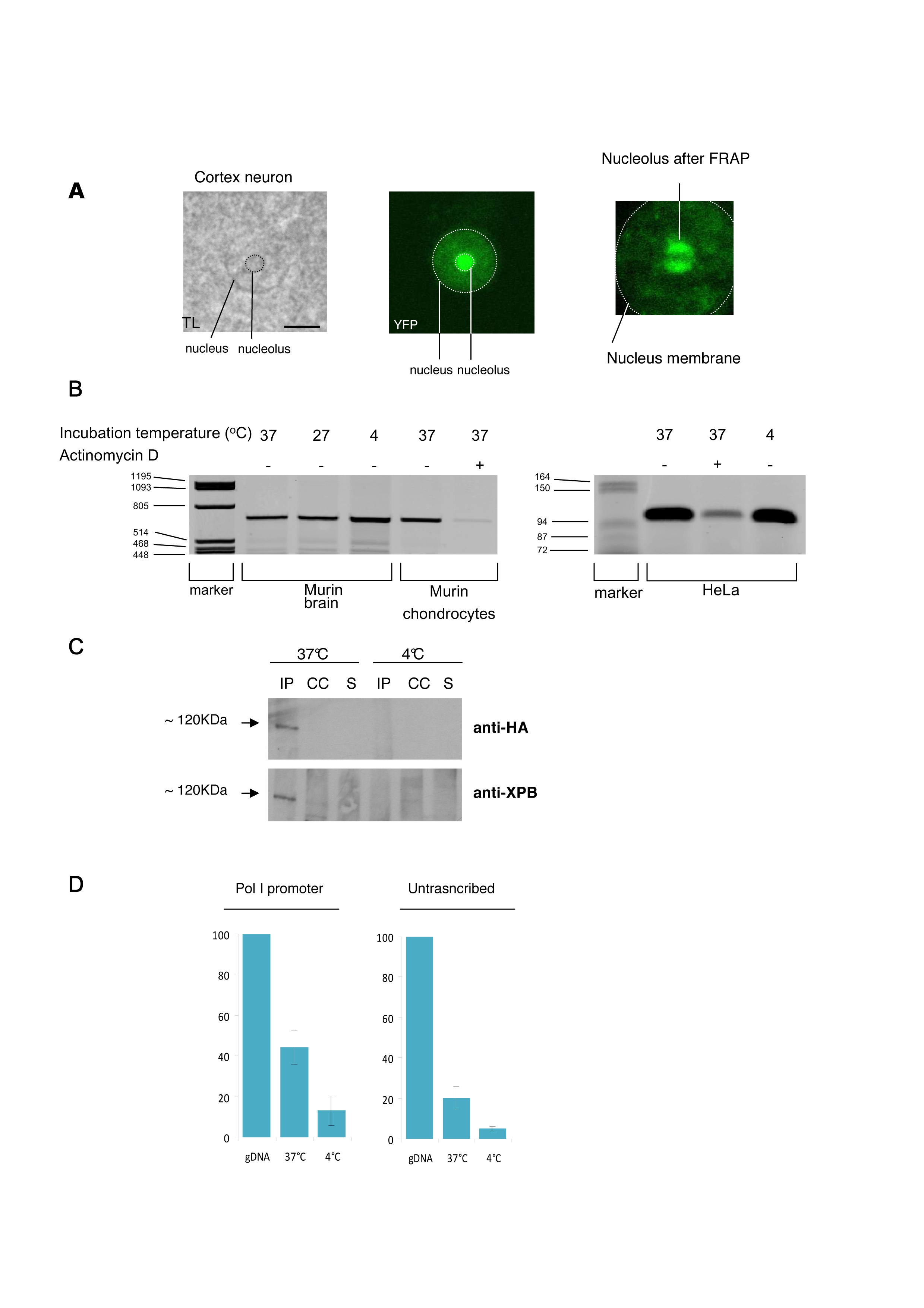

Supplement: Figure S9 — RNAP1 and RNAP2 transcription in neurons. (A) Image of a cortex neuron within organotypic slice in transmitted light (TL) (left panel) and fluorescent light (middle panel). Bar: 10 µm. Left panel: image shows a cortex neuron within an organotypic brain slice 10 min after bleaching part of the nucleolus. (B) Semiquantitative reverse transcriptase (RT)-PCR of pre-rRNA extracted from organotypic cortex slices and cultured chondrocytes (left panel) and Hela cells (right panel), incubated at different temperatures and treated with actinomycin D. (C) Nuclear extract from the brain was immunoprecipitated with HA-antibodies and subjected to Western blot analysis with the indicated antibodies. The input lanes represent Immunoprecipitation fraction (IP), cleared chromatin (CC) cross-linked extract before IP, supernatant (S) of cross-linked extract after IP. (D) Bar graphs representing the quantification of a semiquantitative PCR on 200 ng of genomic DNA (gDNA) and 200 ng of HA-precipitated chromatin from cortex slice incubated at 37°C and 4°C. Promoter sequence from Pol1 gene (−119/+7) and untranscribed region adjacent to the Pol1 gene (Chr 6: 71937600–71938000). The y-axis represents the percentage of amplified sequences from the HA-precipitated chromatin versus amplified sequences from gDNA, set to 100%. Error bars have been calculated for three biological replicates. (0.94 MB DOC) [file pbio.1000220.s009.tif]

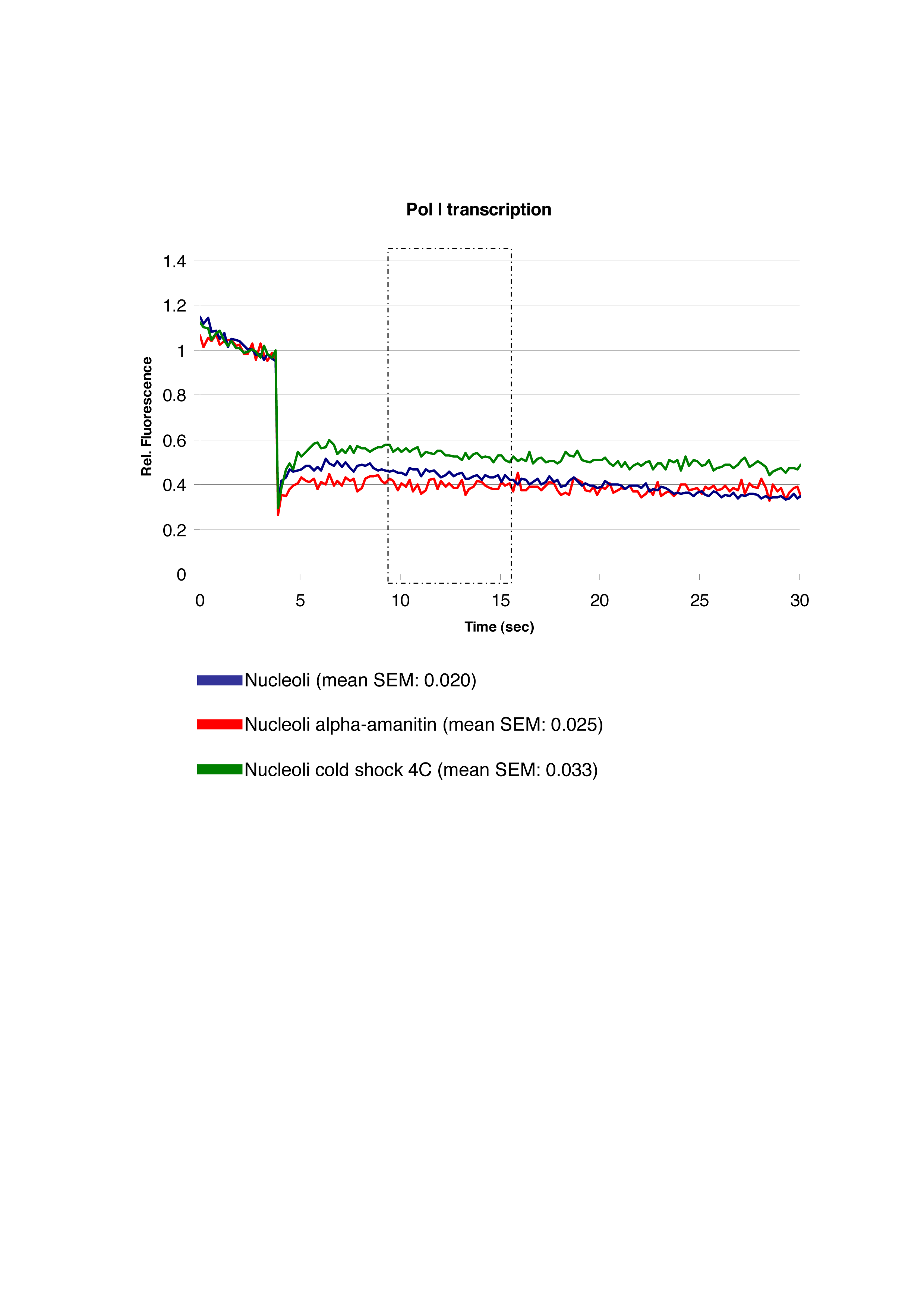

Supplement: Figure S10 — Strip-FRAP graphs used to calculate TFIIH bound fractions indicated in Figure 3A , section Pol 1 transcription. The dotted square indicates the approximate time frame used to calculate the immobile fractions. Mean SEM is the average standard error of the mean calculated over the entire time range of each FRAP curve. (0.23 MB TIF) [file pbio.1000220.s010.tif]

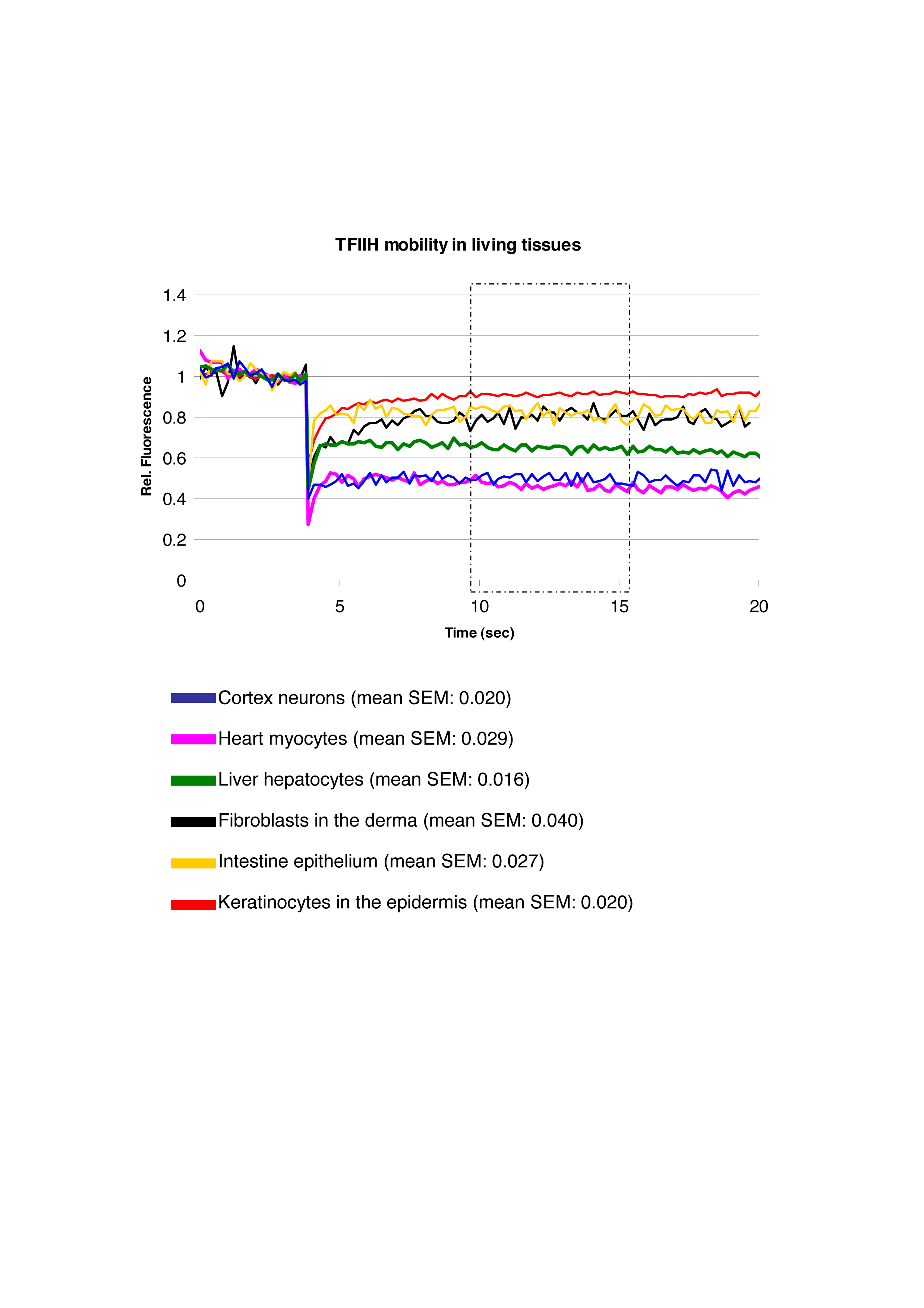

Supplement: Figure S11 — Strip-FRAP graphs used to calculate TFIIH bound fractions indicated in Figure 4A . The dotted square indicates the approximate time frame used to calculate the immobile fractions. Mean SEM is the average standard error of the mean calculated over the entire time range of each FRAP curve. (0.29 MB TIF) [file pbio.1000220.s011.tif]

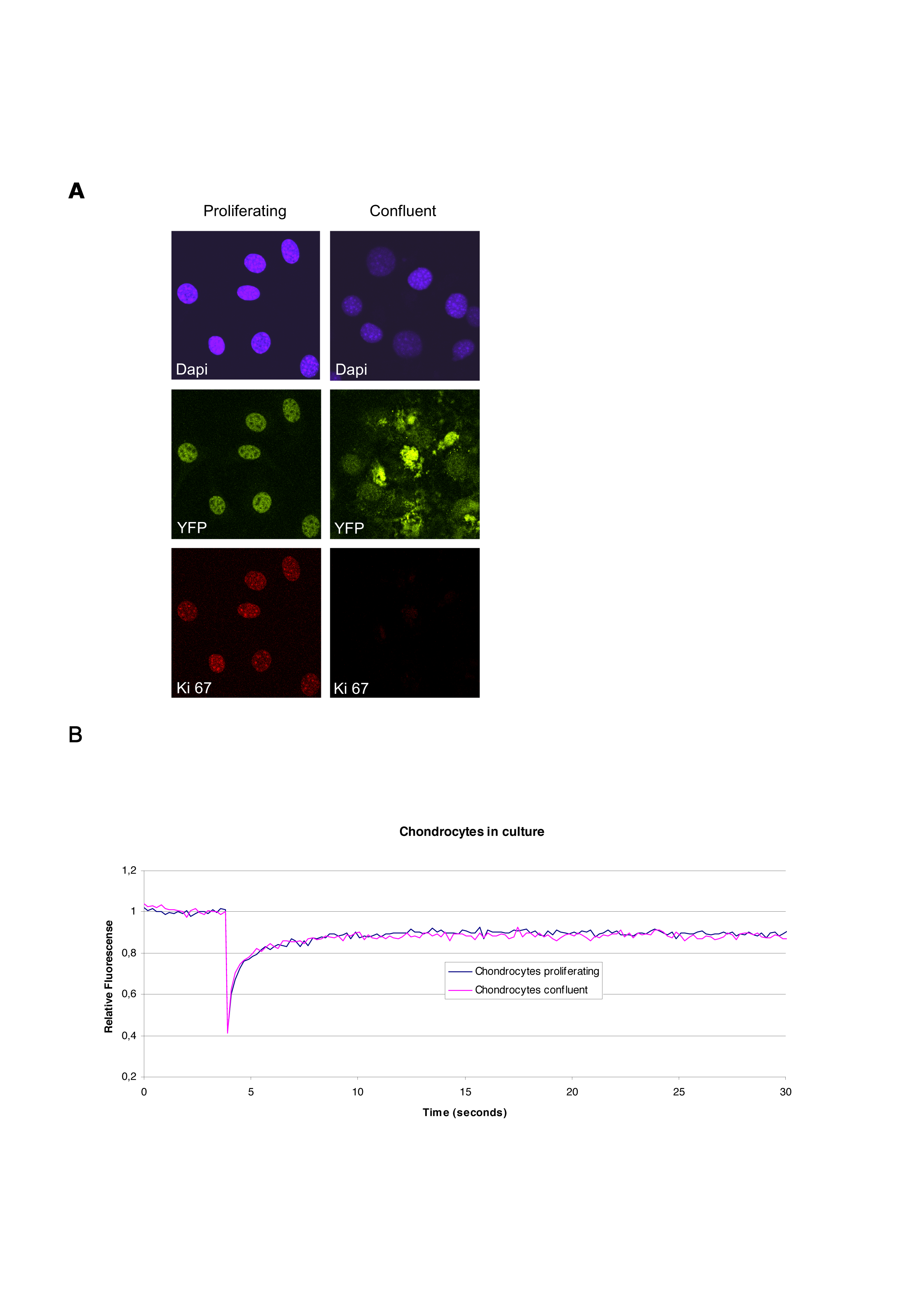

Supplement: Figure S12 — TFIIH mobility in chondrocytes. (A) Confocal imaging of chondrocytes isolated from Xpby/y mice proliferating (left panels) and confluent (right panels). Ki67 immunostaining (in red) was used to confirm the proliferative state of the cells. (B) TFIIH mobility in proliferating (blue) and confluent (red) chondrocytes. (1.34 MB TIF) [file pbio.1000220.s012.tif]

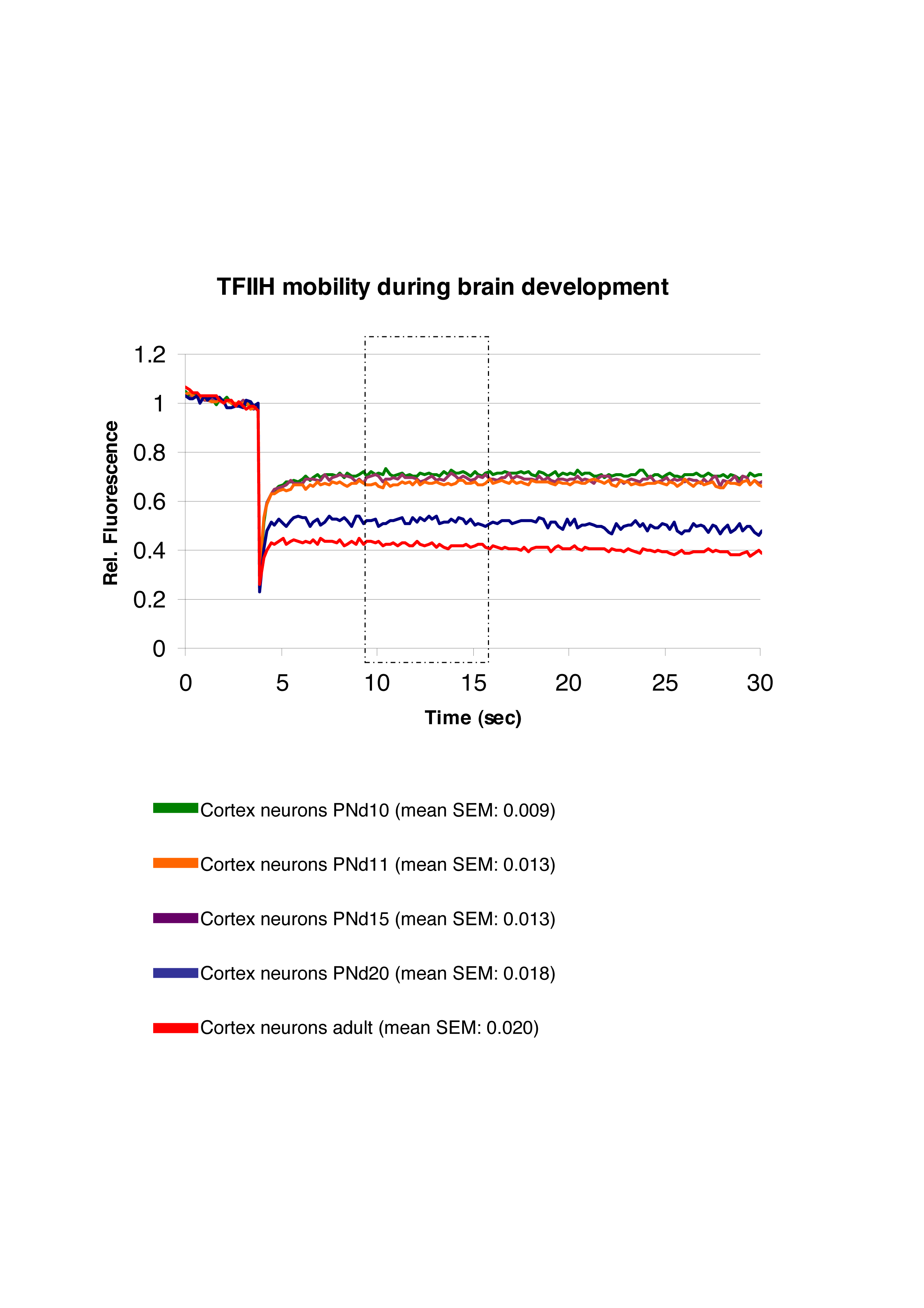

Supplement: Figure S13 — Strip-FRAP graphs used to calculate TFIIH bound fractions indicated in Figure 4B . The dotted square indicates the approximate time frame used to calculate the immobile fractions. Mean SEM is the average standard error of the mean calculated over the entire time range of each FRAP curve. (0.27 MB TIF) [file pbio.1000220.s013.tif]

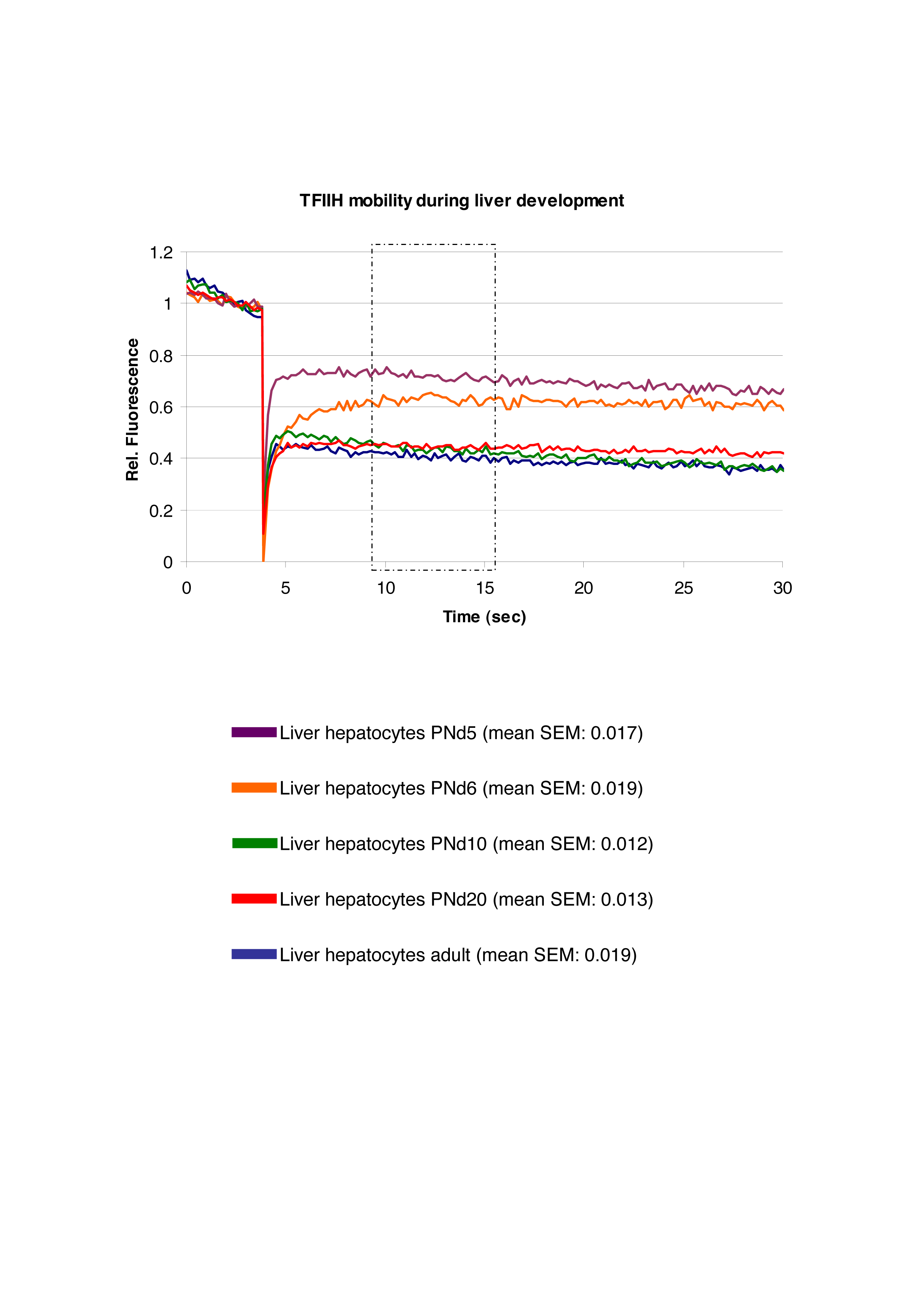

Supplement: Figure S14 — Strip-FRAP graphs used to calculate TFIIH bound fractions indicated in Figure 4C . The dotted square indicates the approximate time frame used to calculate the immobile fractions. Mean SEM is the average standard error of the mean calculated over the entire time range of each FRAP curve. (0.29 MB TIF) [file pbio.1000220.s014.tif]

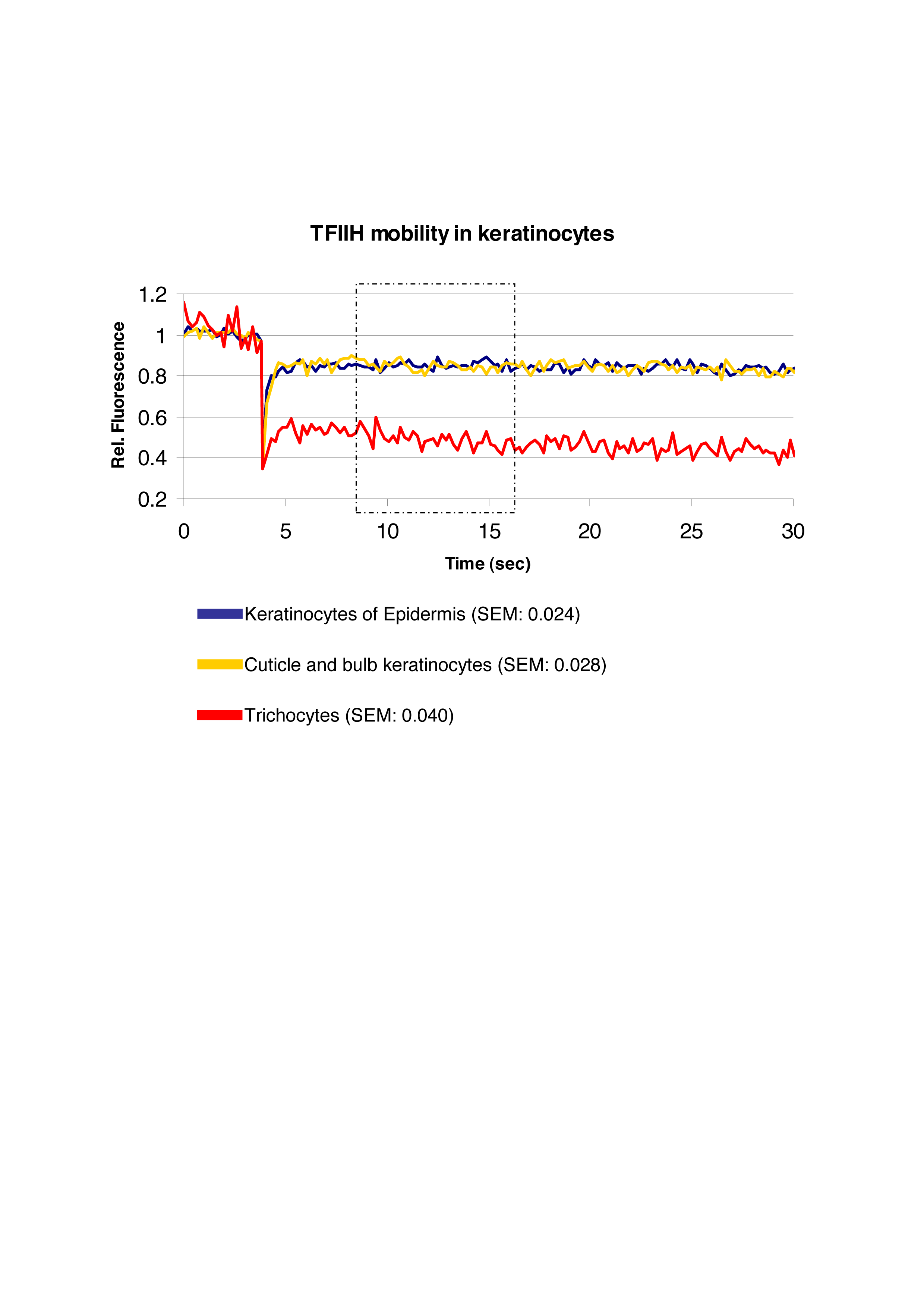

Supplement: Figure S15 — Strip-FRAP graphs used to calculate TFIIH bound fractions indicated in Figure 5B . The dotted square indicates the approximate time frame used to calculate the immobile fractions. Mean SEM is the average standard error of the mean calculated over the entire time range of each FRAP curve. (0.24 MB TIF) [file pbio.1000220.s015.tif]

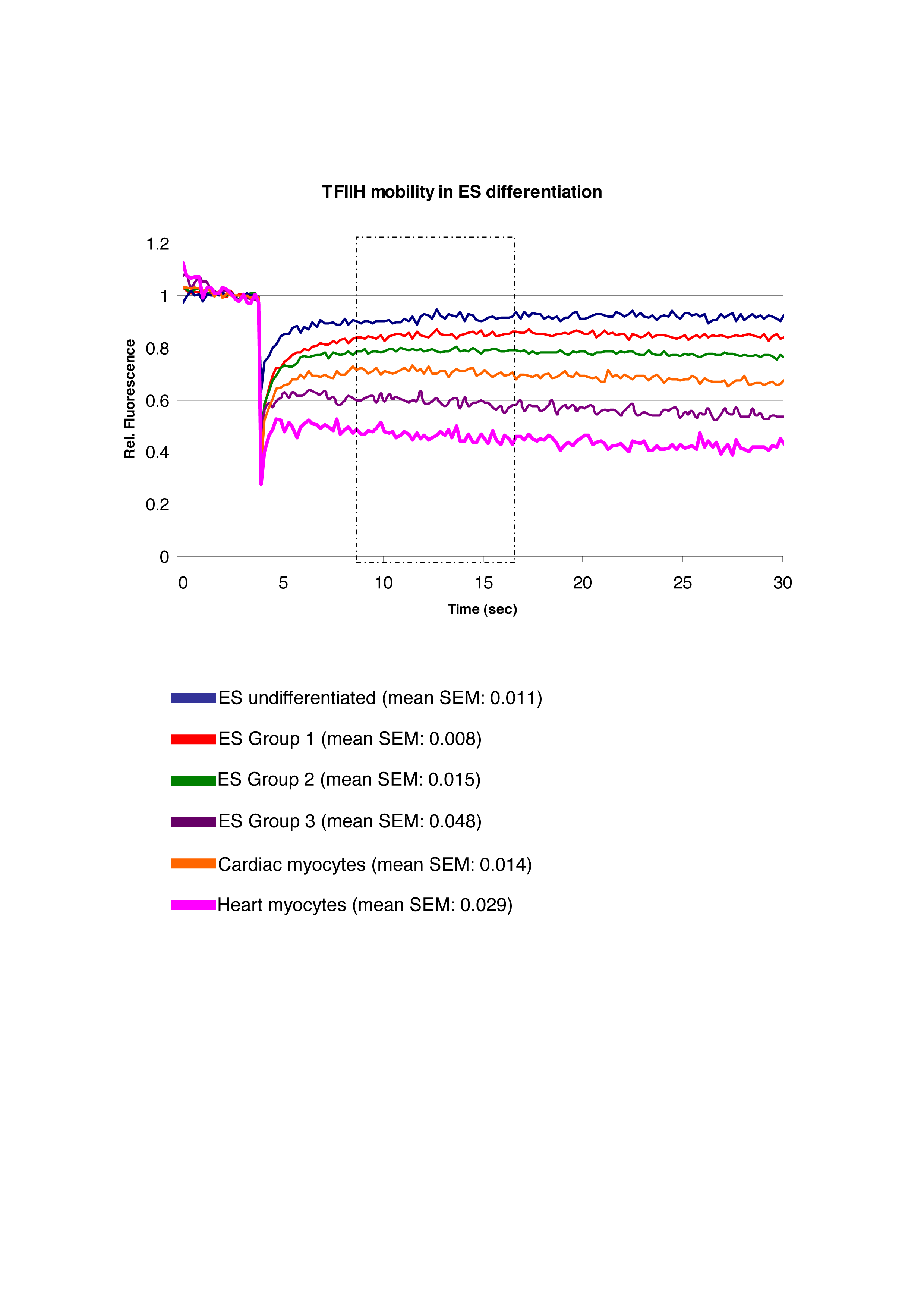

Supplement: Figure S16 — Strip-FRAP graphs used to calculate TFIIH bound fractions indicated in Figure 5C . The dotted square indicates the approximate time frame used to calculate the immobile fractions. Mean SEM is the average standard error of the mean calculated over the entire time range of each FRAP curve. (0.29 MB TIF) [file pbio.1000220.s016.tif]

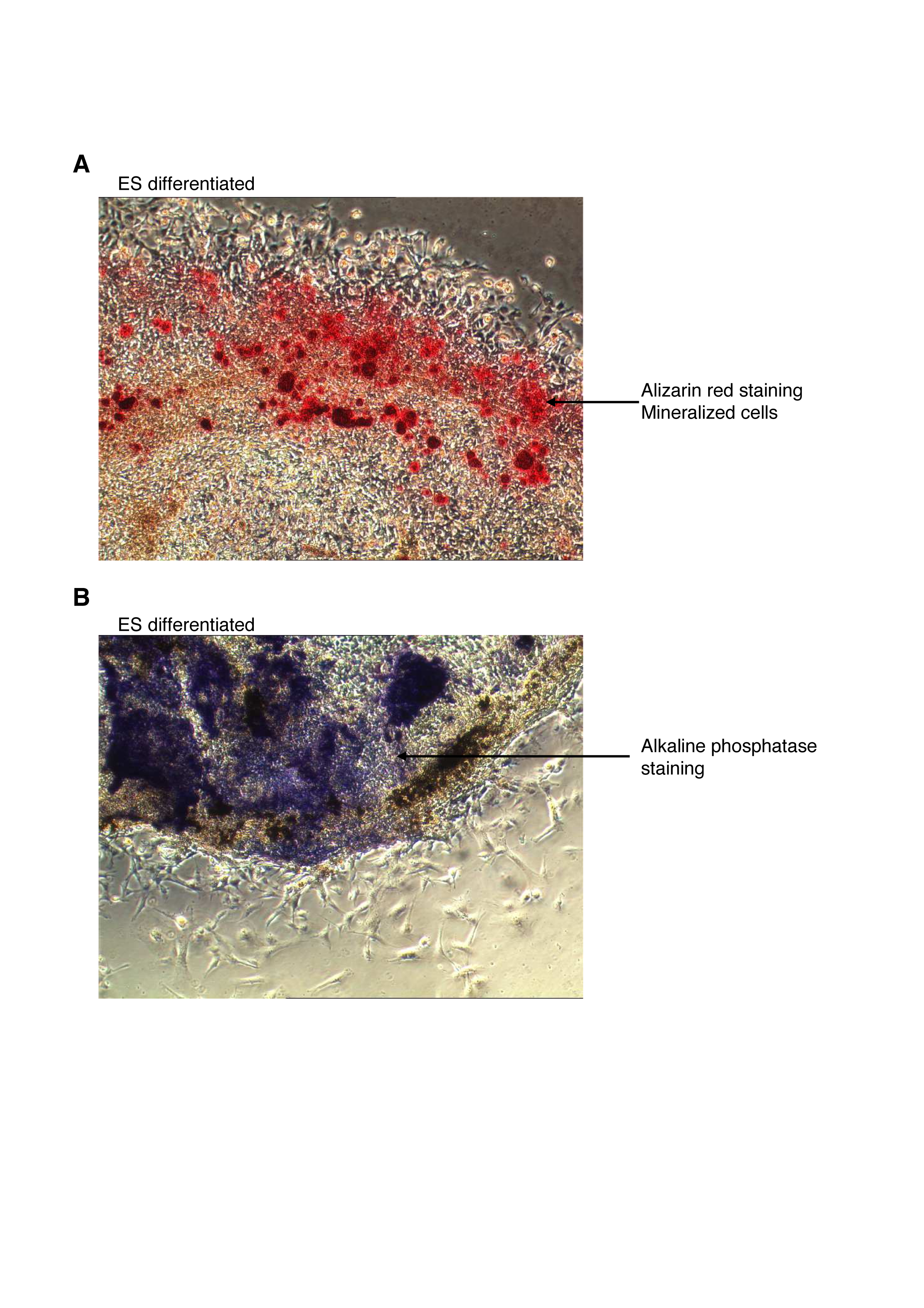

Supplement: Figure S17 — Differentiation markers in ES clones. (A) Transmission light image of a differentiated ES clone stained with Alizarin Red. Red-stained cells and inclusion are characteristically highly mineralized (Ca2+-containing) cells. (B) Transmission light image of a differentiated ES clone stained with alkaline phosphatase. Violet-stained cells are osteocytes producing the enzyme. (5.88 MB TIF) [file pbio.1000220.s017.tif]
